# Supplementary material for: Using digital health tools for the Remote Assessment of Treatment Prognosis in Depression (RAPID): a study protocol for a feasibility study
Source: BMJ Open. 2022 May 6;12(5):e059258. doi: 10.1136/bmjopen-2021-059258 (PMC9083394; doi:10.1136/bmjopen-2021-059258)
Supplement: Supplementary data [file bmjopen-2021-059258supp001.pdf]

SUPPLEMENTARY INFORMATION FOR

Using Digital Health Tools for the Remote Assessment of Treatment Prognosis In Depression (RAPID): A Study Protocol for a feasibility study

Valeria de Angel[1,2], Serena Lewis [1,3], Sara Munir [4], Faith Matcham [1], Richard Dobson [2,5], Matthew Hotopf [1,2]*

1. Institute of Psychiatry, Psychology and Neuroscience, King's College London, London, UK
2. NIHR Maudsley Biomedical Research Centre, South London and Maudsley NHS Foundation Trust, London, UK
3. Department of Psychology, University of Bath, Bath, UK
4. South London and Maudsley NHS Foundation Trust, London, UK
5. Department of Biostatistics and Health Informatics, Institute of Psychiatry, Psychology and Neuroscience (IoPPN), King's College London, 16 De Crespigny Park, London, SE5 8AF, UK

* Corresponding Author contact details:
E3.08, 3rd floor East Wing, Institute of Psychiatry, Psychology and Neuroscience
16 De Crespigny Park, London, SE5 8AF
E: valeria.de_angel@kcl.ac.uk

de Angel V, *et al. BMJ Open* 2022; 12:e059258. doi: 10.1136/bmjopen-2021-059258

Supplementary Note 1 –

Post-Treatment Interview

The primary purpose of this interview is to gain insight into the acceptability of remote assessment and to provide contextual information about participation experiences which may be used to improve future studies using digital sensing technologies.

[TURN ON recording]

Interviewer: "Thank you for agreeing to do the interview. I'd like to ask some questions about your experiences of participating in the study during treatment, specifically, how you found using the technology so the researchers can understand how to deliver a better service in the future. When I talk about participating in the study I mean using the Fitbit, answering Questionnaires on your phone and via email, having the passive app and completing the THINC it app games (the ones about memory and attention). I am keen to understand what worked for you and what **didn't,** so please be completely honest.

This interview is being recorded and should last around 30 minutes, but you can let me know if you would like the recorder to be turned off at any point. All information you provide is anonymous. Information from all the interviews we carry out will be analysed but no identifiable information about you will be included. Also, we might use some quotes from you for academic posters, research papers and a doctoral thesis, but again these will be totally anonymous and not connected to you in any way. Do you have any questions?

This is a semi-structured interview, which means that I have a list of relatively open-ended questions that I will ask you. This means that questions might come up over the course of the interview that you may have already touched on in a previous answer, so If some of the questions seem repetitive or related to your previous answers, please bear with us. We just want to make sure we understand your experience the best we can - thank you!"

A) Participation experience: Identify opportunities for improvement in the study experience (3 mins)

1. How are you finding the experience of participating?
2. Are you finding any aspect of participating in the study particularly burdensome? Can you give me some examples of how/when/why? Anything else that you dislike?

Prompts: [Remind them that this isn't just about the Fitbit] Do you ever feel you needed to spend more time or effort than you would like to? Does taking part cause any disruption to your life?

3. Do you see any personal benefits of participating in the study? Can you give me a few examples of what they are? Anything else that you particularly like.

Prompts: remind them that this isn't just about the Fitbit.

4. Did the study change the way you monitored your health in any way?

B) Working the Study into Daily Life: Identify pain points of fitting the study into daily life (3 mins)

5. Has taking part in this study fit into your usual routine?

Prompts: Did you have to change any aspect of your routine in order to be able to take part? What didn't fit?

de Angel V, *et al. BMJ Open* 2022; 12:e059258. doi: 10.1136/bmjopen-2021-059258

6. Are there any aspects of the study that make it difficult for you to fully participate?

Prompt: Did anything stop you from completing some of the tasks/questionnaires or wear the Fitbit?

7. What could have made it easier for you to participate in this study?
8. Did tracking your behaviour change your behavior?

Prompt: Would you have behaved differently if you had not been wearing a Fitbit, or if you knew your data/responses wouldn't eventually be analysed by a research team.

C) Participation during Psychological Treatment (12 mins)

9. If you have experienced changes in your mood or anxiety levels in the past few months, has it affected the way you have participated in this study? In what way?

Prompts: Has an improvement in mood made you more likely to engage with some of the tasks/questionnaires? Was it easier to remember or motivate yourself to continue?

10. Do you feel like being in this study has had an impact on the psychological therapy you have received? In what way?

Prompt: Has it made you more/less likely to attend sessions, more/less likely to engage with homework. Did tracking your behaviour, sleep, etc impact how you experienced therapy sessions?

11. Did you talk to your therapist about the study? In what way? What about it?

12. Was participating in this study <u>during treatment</u> a positive, negative. neutral experience for you? In what way?

13. If so, what sorts of things do you think could be done to increase any positive impact it has on your treatment.

Prompt: Is there any way in which the wearables or questionnaires could be used in a way which complements therapy within IAPT?

14. What could be done to decrease any negative impact on your treatment?
15. You've been in the study before, during, and after treatment. Was it easier/harder to participate in the study <u>during</u> treatment than outside of it?

16. Do you feel like tracking your behaviour, mood, sleep has had an impact on your mental health?

17. How did you feel about receiving treatment remotely (via telephone or video call)?

Prompt: was it easy/difficult to communicate? Were you comfortable with the technology? Was it convenient?

D) Experience with Apps and wearables: Assess familiarity, learning curve, pain points (4 mins)

18. Had you ever used health tracking apps before coming into the study? What for?

Prompts: by health apps I mean apps that you use to measure or help with physical health/activity or mental health, e.g., Strava, headspace other anxiety apps, food intake, dairies, etc.

19. Were you familiar with wearable devices (activity tracker, health tracker, etc.) prior to this study?

Prompts: What type of device was it? How long did you use it? If you stopped using it, why did you stop?

de Angel V, *et al. BMJ Open* 2022; 12:e059258. doi: 10.1136/bmjopen-2021-059258

20. Was there anything else you would have liked to keep track of during treatment?
Prompts: anything to do with physical health, or emotional/mental health, diaries, etc.

21. Do you ever have any difficulties using the device? Can you give me some examples?
Prompts: At night, for exercise, During charging? During syncing? During setup? Were you able to wear the device for long periods of time or whilst you were sleeping without it causing any annoyance or discomfort?

22. Have you ever chosen not to wear the device? Why was that, and why/when did you start wearing it again?
Prompts: Did level of comfort impact on your choice to wear the device?

23. Are you ever concerned about what people thought of you wearing the device? Can you give me an example?
Prompts: Did it make it easier or harder for you to take part in this study? Did it make you more or less concerned about what others would think about you using the smartphone or wearable device?

24. Because of the pandemic and the social distancing measures taken by the government, do you feel differently about using smartphones and wearable devices? Can you tell me why?
Prompt: do you feel more, or less, inclined to use and engage with your smartphone or wearable devices?

E) Data Collection, Privacy and Sharing (4 mins)

25. How did you feel about the data being collected passively/automatically, without you needing to interact with the device?
Prompt: Did you like this more or less than when you had to interact with the device, e.g. when you had to complete a survey?

26. How would you feel about the information collected during the study going to your therapist or healthcare provider?
Prompts: What are your thoughts about the data being automatically available to your GP or healthcare provider? Would you like to be able to choose what information they receive?

27. Would it have been useful for you to receive information about the data collected throughout the study? Why or why not?
Prompts: Would it make you more or less likely to participate in a future study? If you would look at feedback, how would you like to receive this? Should it be optional? Provided in the app? Should it be visual, with graphs, or verbal feedback? What kinds of graphs are useful? How often should it be provided?

28. Did you ‘feel monitored’?
Have the impression of being watched or that your movements were being tracked, monitored.

F) Closing & Improvements needed in Study Design (1 mins)

29. Do you think there is value in this approach to gathering data about your health? How would you describe that value?

30. Is there anything else you would like to say?
