# Supplementary material for: Using digital health tools for the Remote Assessment of Treatment Prognosis in Depression (RAPID): a study protocol for a feasibility study
Source: BMJ Open. 2022 May 6;12(5):e059258. doi: 10.1136/bmjopen-2021-059258 (PMC9083394; doi:10.1136/bmjopen-2021-059258)
Supplement: Author's manuscript [file bmjopen-2021-059258.draft_revisions.pdf]

BMJ Open

BMJ Open is committed to open peer review. As part of this commitment we make the peer review history of every article we publish publicly available.

When an article is published we post the peer reviewers' comments and the authors' responses online. We also post the versions of the paper that were used during peer review. These are the versions that the peer review comments apply to.

The versions of the paper that follow are the versions that were submitted during the peer review process. They are not the versions of record or the final published versions. They should not be cited or distributed as the published version of this manuscript.

BMJ Open is an open access journal and the full, final, typeset and author-corrected version of record of the manuscript is available on our site with no access controls, subscription charges or pay-per-view fees (http://bmjopen.bmj.com).

If you have any questions on BMJ Open's open peer review process please email info.bmjopen@bmj.com

BMJ Open

Using Digital Health Tools for the Remote Assessment of Treatment Prognosis In Depression and Anxiety (RAPID): A Study Protocol for a feasibility study

Journal:	*BMJ Open*
Manuscript ID	bmjopen-2021-059258
Article Type:	Protocol
Date Submitted by the Author:	15-Nov-2021
Complete List of Authors:	de Angel, Valeria; King's College London, Department of Psychological Medicine<br>Lewis, Serena; King's College London; University of Bath, Department of Psychology<br>Munir, Sara; South London and Maudsley NHS Foundation Trust<br>Matcham, Faith; King's College London, Department of Psychological Medicine, IoPPN<br>Dobson, Richard; King's College London, Department of Biostatistics and Health Informatics; South London and Maudsley NHS Foundation Trust, NIHR Maudsley Biomedical Research Centre<br>Hotopf, Matthew; Institute of Psychiatry Psychology and Neuroscience Division of Academic Psychiatry
Keywords:	Depression & mood disorders < PSYCHIATRY, Anxiety disorders < PSYCHIATRY, MENTAL HEALTH, Health informatics < BIOTECHNOLOGY & BIOINFORMATICS

SCHOLARONE™
Manuscripts

Using Digital Health Tools for the Remote Assessment of Treatment Prognosis In Depression and Anxiety (RAPID): A Study Protocol for a feasibility study

Valeria de Angel[1,2], Serena Lewis [1,3], Sara Munir [4], Faith Matcham [1], Richard Dobson [2,5], Matthew Hotopf [1,2]*

1. Institute of Psychiatry, Psychology and Neuroscience, King's College London, London, UK
2. NIHR Maudsley Biomedical Research Centre, South London and Maudsley NHS Foundation Trust, London, UK
3. Department of Psychology, University of Bath, Bath, UK
4. South London and Maudsley NHS Foundation Trust, London, UK
5. Department of Biostatistics and Health Informatics, Institute of Psychiatry, Psychology and Neuroscience (IoPPN), King's College London, 16 De Crespigny Park, London, SE5 8AF, UK

* Corresponding Author contact details:
E3.04, 3rd floor East Wing, Institute of Psychiatry, Psychology and Neuroscience
16 De Crespigny Park, London, SE5 8AF
E: Valeria.de_angel@kcl.ac.uk

Word count:3863

Key words: Depression, Anxiety, Psychological therapy, Feasibility, Remote measurement technology, Passive sensing, mhealth, eMental health, digital phenotype, digital health tools, prospective cohort

For peer review only

For peer review only - http://bmjopen.bmj.com/site/about/guidelines.xhtml

Abstract:

Introduction:

Digital health tools such as smartphones and wearable devices could improve psychological treatment outcomes in depression through more accurate and comprehensive measures of patient behaviour. However, the field is in its infancy, with the literature comprised of mostly small-scale studies with student populations outside of a clinical setting. The current study aims to determine the feasibility and acceptability of using smartphones and wearable devices to collect behavioural and clinical data in people undergoing therapy for depressive disorders and establish the extent to which they can be potentially useful biomarkers of depression and recovery after treatment.

Methods and analysis:

This is an observational, prospective cohort study of 65 people attending psychological therapy for depression and anxiety in multiple London-based sites. It will collect continuous passive data from smartphone sensors and a Fitbit fitness tracker, and deliver questionnaires, speech tasks and cognitive assessments through smartphone-based apps. Objective data on sleep, physical activity, location, Bluetooth contact, smartphone use, and heart rate, will be gathered for 7 months, and compared to clinical and contextual data. A mixed methods design, including a qualitative interview of patient experiences, will be used to evaluate key feasibility indicators, digital phenotypes of depression and anxiety, and therapy prognosis. Public and Patient Involvement was sought for participant-facing documents and the study design of the current research proposal.

Ethics and dissemination.

Ethical approval has been obtained from the London Westminster Research Ethics Committee, and the Health Research Authority, IRAS project ID: 270918. Privacy and confidentiality will be guaranteed and the procedures for handling, processing, storage and destruction of the data will comply with the General Data Protection Regulation (GDPR). Findings from this study will form part of a doctoral thesis, will be presented at national and international meetings or academic conferences, and will generate manuscripts to be submitted to peer-reviewed journals.

Registration details: https://doi.org/10.17605/OSF.IO/PMYTA

Strengths and limitations of this study:

- Remote measurement technologies are being used in a clinical context, and therefore have the potential to inform implementation efforts within mental health services.
- The study has the potential to uncover digital signals of improvement related to psychotherapy, which could help early identification of non-responders to treatment.
- A 7-month participant follow-up provides a picture of engagement in the longer-term, compared to previous studies.
- Qualitative and quantitative approaches will be combined to evaluate feasibility and acceptability.
- For pragmatic reasons, the study uses a non-randomised design, which will limit conclusions about digital changes to treatment response.

For peer review only

For peer review only - http://bmjopen.bmj.com/site/about/guidelines.xhtml

INTRODUCTION

Depression is a leading cause of disability worldwide [1], yet response to treatment is poor, with only 50-60% of people recovering after 3 months of treatment [2,3]. Mental health science relies almost exclusively upon subjective self-report to diagnose mental illness and measure outcomes. This subjective data means reliance is placed on patients being able to accurately recall and communicate complex mood states during clinical interviews, which many patients find difficult [4,5]. Subjective data potentially introduces recall biases [6] which may worsen with increased severity [7].

The use of digital tools within mental health has the potential to enhance these traditional self-report measures by improving aspects of symptom tracking, illness management and treatment support. Remote measurement technologies (RMTs) such as smartphones and wearable devices can unobtrusively capture a more accurate picture of a patient's clinical state in a continuous way, with far less burden to the user. Through embedded sensors, they can detect changes in behaviours associated with depressive symptomatology such as sleep [8], sociability [9], physical activity [10], and speech [11]. Detecting such changes in a person's behaviour can provide invaluable information for tailoring and improving treatment.

Given the relative recency of the field, and with an eye towards clinical implementation, studies on RMTs and depression have largely comprised proof-of-concept, feasibility, and acceptability studies. While studies show RMTs to be generally feasible and acceptable [12], the data predominantly come from small, non-clinical or student samples with a median follow- up time of two weeks[13].

To our knowledge, no studies have been published on the use of RMTs to track mood and behaviour in clinical populations undergoing psychotherapy for depression or anxiety disorders. This population could derive greater benefits from the application of such digital methods by alleviating distress in more potentially severe cases and reducing pressure on healthcare services. In addition to the barriers of adopting RMTs for mood monitoring found across populations, such as concerns around privacy, confidentiality, affordability and accessibility, there are likely to be additional considerations related to the help-seeking populations that remain unexplored and may help or hinder implementation practices in the future.

From a clinical perspective, exploring feasibility and acceptability in therapy populations can shed light on how changes in severity affect engagement with and use of digital devices, how they can be used to complement treatment, and the barriers and facilitators to their use and implementation within services. From a methodological perspective, it would be important to establish the extent to which the amount and quality of data is usable and unbiased given that the added workload from therapy exercises, homework and clinical questionnaires as well as the severity of symptoms such as decreased motivation and cognitive abilities, are likely to affect engagement and device use [12]. Such studies could also inform future projects about the likely uptake in these samples in order to establish sample sizes and allocate resources.

Study aims:
The primary aim of this project is to determine the feasibility of using RMTs, such as Android smartphones and a Fitbit wearable device, to collect behavioural and clinical data in people undergoing therapy for depressive disorders, to establish the extent to which they can be potentially useful biomarkers of depression and changes in clinical state.

Secondary aims are to identify candidate signals for digital biomarkers by detecting correlations between objective features and clinical characteristics, and to explore whether these signals have prognostic value in the context of psychological treatments.

METHODS AND ANALYSIS

Design
This is an observational, prospective cohort study of people attending psychological therapy for depression and anxiety. It will use RMTs to gather active and passive data for 5 - 7 months and will adopt a mixed methods design to evaluate the feasibility and acceptability of such data collection methods.

Setting
Participants will be drawn from Improving Access to Psychological Therapies (IAPT) services in South London. The participating IAPT services will be from the boroughs of Lambeth, Lewisham and Croydon within the South London and Maudsley NHS Foundation Trust. IAPT is a publicly funded self-referral outpatient programme providing evidence-based psychological treatments for adults with depression and anxiety disorders. The service is free at the point of delivery.

For peer review only - http://bmjopen.bmj.com/site/about/guidelines.xhtml

The current study begun recruitment in June 2020, during the COVID-19 pandemic. Given the government-imposed travel restrictions and social distancing measures, the entirety of this study will be carried out remotely.

Sample size

Formal sample size calculations are not a requirement for feasibility studies [14], however, the general recommendation is for samples of 50-60 participants to assess feasibility outcomes [15]. In order to address our secondary aims, a sample size of 50 would be sufficiently powered to detect a correlation coefficient of 0.39 and above, assuming a significance level of 0.05 and Type 2 error value of 0.20. Based on previous studies, we expect such an effect size [16–18]. To account for a potential attrition rate of 20% we will aim to recruit 65 participants.

Recruitment

IAPT clinicians will act as gatekeepers to the initial recruitment process. Patients within their service, who have previously agreed to be contacted for research purposes, will be invited to take part, either by phone call or email. They will be given a summary of the aims and procedures of the study and be screened for eligibility as per the inclusion/exclusion criteria below. When screening is done by email, participants receive a personalised email with a description of the study and a link to an online screening tool that participants can complete in their own time, the responses of which are relayed to the research team. If willing and eligible, potential participants will be sent the participant information sheet and given at least 24 hours before going through the consent procedures and being enrolled in the study.

Inclusion criteria:

a) Adults with a current depressive episode as measured by The Mini International Neuropsychiatric Interview (MINI; [19]
b) Being on the waiting list to receive treatment for depression or anxiety disorders at IAPT services, with an expected wait of at least 7 days (to a maximum of 5 weeks) between scheduled enrolment and first treatment session.
c) Existing ownership of Android smartphone with sufficient memory space for the relevant apps.
d) Able and willing to use a wrist worn device for duration of the study
e) Able to give informed consent for participation.
f) Sufficient English language skills to understand consent process and questionnaires.

Exclusion criteria:

a) Lifetime diagnosis of bipolar disorder, schizophrenia and schizoaffective disorders as these have different digital patterns to depression [20,21]
b) Health anxieties that may significantly worsen with constant monitoring of behaviour.
c) Extensive sharing of smartphone with friends or family
d) Night shifts, pregnancy, or living with a 0–6-month-old baby (due to sleep disruptions)

Study procedures

Once interest and eligibility have been ascertained, participants will be invited to attend an enrolment session via videocall. Figure 1 shows the study timeline for participants as they enter the study. After a further review of study procedures and opportunity for questions, participants will be asked to sign an electronic consent form. Consent can be taken either using the Qualtrics platform, which has been approved for this purpose, or via MS Word or PDF documents which participants electronically sign from their devices.

Enrolment/Baseline

Following consent, the enrolment session is comprised of three further sections: (1) obtaining sociodemographic and clinical data, (2) completion of self-reported questionnaires, and (3) technology set up. The researchers will take demographic and clinical information related to current and previous physical and mental health conditions, family history, treatment status as well as phone use, social and physical activity levels. In order to detect the presence of a depressive episode, and define whether atypical in nature, MINI and the Atypical Depression Diagnostic Scale (ADDS) [22] will be administered. At the end of the session, participants will be asked to complete a battery of self-reported questionnaires as shown in Table 1.

Technology set-up

Participants will be asked to download 4 apps on their phone; RADAR passive RMT (pRMT) app, which collects background sensor data from smartphones; the RADAR active RMT (aRMT) app, which delivers clinical questionnaires; THINC-it® for RADAR-CNS, an app assessing cognitive function; and the Fitbit app. These will be linked in-call to the RADAR-base platform [23]. A Fitbit Charge 3 or 4 is then delivered to them within 1-2 working days, at which point they are guided through the set up. Participants will be given £10.00 for completing the enrolment session and keep the Fitbit after the study. Table 1 shows the schedule of events for the RAPID study.

For peer review only - http://bmjopen.bmj.com/site/about/guidelines.xhtml

For peer review only

Follow-up:
From enrolment, longitudinal collection of active and passive data begins. The current study will use the RADAR-base platform and their apps to collect passive and active data, as well as a Fitbit API integration source [23]. More information can be found at radar-base.org/.

Passive Measures:
Passive measures will be continuously gathered from smartphone sensors via the pRMT app and wearable sensors from the Fitbit. Sensor data will include GPS, acceleration, step count, light, phone interaction (total time on phone and app usage), paired and nearby bluetooth devices, number of saved contacts, battery level and weather. Fitbit generates digital features relating to sleep, physical activity and heart rate.

No personally identifiable information will be gathered from these sensors, and no contact details nor website or app content is collected by the apps. Personal privacy is thus protected, and no identification of an individual's home address or precise geographical location can be gathered.

Active measures:
Participants will be asked to respond to questionnaires throughout the study period. Some of them will be delivered via the aRMT app, others will be collected via the Research Electronic Data Capture (REDCap) software [24], a web-based platform for research that sends e-mail notifications to participants throughout the study.

(a) Weekly emailed questionnaires: Participants will receive weekly emails with a link to complete REDCap-delivered questionnaires, which can be complete on a smartphones or a computer. Questionnaires will be scheduled at different time intervals (fortnightly, monthly), in such a way that the maximum amount of time needed to complete them is 10 minutes per week.
(b) Weekly aRMT tasks: The aRMT app is designed to collect health information from research participants by sending them notifications and asking them to complete in-app tasks and questionnaires. These will include questions on depression, functionality, subjective sleep experiences and a speech task.
(c) Speech Task: Participants will be asked to undertake 2 speech tasks. The first task will require them to read out pre-written text, and the second task will ask them to answer out loud a question such as: "Can you describe something you are looking forward to this week?". Participants will record their voice in quiet surroundings for both tasks via the aRMT app. Acoustic features such as pitch, jitter, shimmer, formants and intensity will be extracted.
(d) Cognition via THINC-it® app: Once a month, the aRMT app will notify participants that it's time to complete the THINC-it tasks, they will be asked to open the THINC-it app to do so. THINC-it is a validated tool designed to assess cognitive function in depression [25]. The tests incorporated in this tool – the One-Back Test, the Trail Making Test Part B, the Digit Symbol Substitution Test, Choice Reaction Time task – assess attention, processing speed, executive function, learning and memory. The tool also incorporates the Perceived Deficits Questionnaire [26], a self-report questionnaire that assesses a person's cognitive concerns.

Table 1. Schedule of events.

Event	Enrolment	Questionnaire Frequency			
		Weekly	Fortnightly	Monthly	Endpoint
Informed Consent	x				
Sociodemographics	x				
Clinical History	x				
MINI	x				
ADDS	x				
Smartphone apps set-up	x				
**Active Measures[a] (from REDCap)**					
Validated Questionnaires:					
SAPAS	x				
BIPQ	x				x
Life stress (SRRS)	x				x
CTQ	x				
AUDIT	x				

For peer review only - http://bmjopen.bmj.com/site/about/guidelines.xhtml

PHQ – 9	x		x		x
GAD-7	x		x		x
Rumination (RRS)	x			x	
AUDIT (short version)	x		x		
Oslo 3 Social support	x			x	
Perceived Stress Scale	x			x	x
WAI – SR**					x
Contextual information:					
Caffeine Intake	x	x			
Treatment status and content*	x	x			
Social activities	x		x		
Social distancing practices	x		x		
COVID experience	x			x	
**Active Measures[a] (from aRMT app):**					
(Q)IDS – SR	x	x			
WSAS	x	x			x
Speech Task	x		x		
Perceived sleep[ɪ]	x	x			
Cognition (THINC-it app)	x			x	
**Passive Measures:**					
Fitbit Charge	To be worn throughout the study.				
pRMT app	Will run in the background gathering data from enrolment.				
**Qualitative Interview****					x

[a] Weekly time spent completing questionnaires should not exceed 10 mins.
* Only for the duration of treatment.
** Completed once during treatment
[ɪ] Daily for 90 days.

End of study:
Twelve weeks after participants have finished treatment, the research team will contact them to finalise their time in the study and complete endpoint assessments. In case of an unexpected change regarding their treatment, such as treatment being reduced or extended, the 3-month follow-up will commence on the day of their last core treatment session with IAPT services.

Extra Participant contact:
To maintain engagement and stay abreast of any issues, participants will be contacted after their first week post enrolment, and then in months 1, 3, 5 and 7. Researchers will initially contact participants on the phone unless an alternative method of contact is preferred and followed up with an email. Any issues raised in these calls, or sporadically reported by participants throughout the course of the study will be recorded. Additionally, participants will be sent a monthly newsletter, via e-mail, which will include study updates, tech tips and any frequently asked questions.

Post-treatment Qualitative Interview:
In order to inform the feasibility and acceptability aims of this study, participants who complete therapy will be invited to take part in an optional qualitative interview. It will be a 30-minute semi-structured interview looking at participant experiences of using RMTs during psychotherapy for depression. We will invite participants to this interview once they complete treatment and will interview the first 20 who agree to take part.

Outcome measures

Primary outcomes

The primary outcome is to establish the feasibility of using wearable devices and smartphone sensors to monitor the behaviour of people with depression whilst receiving psychological treatment. Key feasibility outcomes will be related to clinical and methodological considerations, and will evaluate recruitment and participant flow, subjective reports of acceptability of methods, data availability and data quality.
The following feasibility outcomes will be reported:

(1) Estimates of recruitment and attrition rates (Figure 2)

For peer review only - http://bmjopen.bmj.com/site/about/guidelines.xhtml
For peer review only

(2) Presence and absence of passive data: 'wear time' for wearable devices and 'on time' for smartphone sensors, and the amount of data necessary to conduct correlational and predictive analyses.
(3) Active data availability and data quality: percentage number of tasks completed.
(4) Qualitative data: Participant experience and attitudes towards data collection instruments and procedures.

Secondary Outcomes

Secondary outcomes will evaluate the relationship between digital data and clinical outcome measures, both at individual time points and as prognostic factors for recovery after treatment. Scores on clinical scales will be used in addition to individual symptom domains.

Clinical state will be measured using:

- Patient Health Questionnaire (PHQ – 9) [27]: the 9-item questionnaire that is widely used for measuring depression in IAPT services.
- Generalised Anxiety Disorder questionnaire (GAD-7) [28]: will be measured as it gathers important anxiety symptoms which are so often comorbid with depression.
- Quick Inventory of Depressive Symptomatology – Self-rated (QIDS – SR)[29]: This is a 16-item inventory of depression for patients who identify as depressed or who may be suffering from depression.
- Work and Social Adjustment Scale (WSAS)[30]: a measure of quality-of-life/disability, which is a 5-item assessment of perceived social and work-related functional impairment used widely across a range of mental and physical disorders.
- Cognition via the THINC-it app.

Participants will be considered to be in remission if they have experienced a reduction of at least 50% in depressive symptomatology from the start of treatment, or no longer meet criteria for depression according to the PHQ-9 (scoring below the cut-off of 5). Sub-dimensions of depression – for example interest in activities, motivation, appetite – will be gathered from the QIDS-SR.

The socio-demographic, clinical and contextual variables measured, such as illness severity, cognitive function and social support will be taken as covariates.

- Standardised Assessment of Personality: Abbreviated Scale (SAPAS) [31] – an 8-item personality test that screens for personality disorder.
- Brief Illness Perceptions Questionnaire [32]. The BIPQ provides an insight into the participant's views about their underlying condition and how well they see themselves coping with it.
- Life stress scale (SRRS) [33] – this is a retrospective questionnaire for identifying major stressful life events.
- Childhood Trauma Questionnaire (CTQ) [34] – a 26-item scale that assesses five types of maltreatment; sexual abuse, physical abuse, emotional neglect, physical neglect and emotional abuse.
- Alcohol Use Disorder Identification Test (AUDIT) [35] – widely used scale in primary care that measures alcohol consumption, drinking behaviours and identifies harmful alcohol use.
- Oslo 3-items social support scale (OSSS – 3) [36] – is a brief instrument that assesses social support.
- Perceived Stress Scale (PSS) [37] – It is a measure of the degree to which situations in one's life are appraised as stressful. Items were designed to assess how unpredictable, uncontrollable, and overloaded respondents find their lives to be.
- Working Alliance Inventory – Short-Revised (WAI-SR) [38]: a 12-item scale measuring therapeutic alliance on 3 key components: (a) agreement on the tasks of therapy, (b) agreement on the goals of therapy and (c) development of an affective bond.
- Rumination Response Scale (RRS) [39] – a 10 item scale that measures rumination.
- COVID related questions – isolation status and their perceived effect, confirmed or suspected diagnosis of COVID19 as well as social distancing practices.
- Treatment status – questions on whether psychotherapy has begun, whether they take concomitant medication and adherence (e.g. "Are you taking medication for your mental health? If so, which one?" or "Have you missed any psychotherapy sessions this week?"), and the broad content of their therapy sessions.

STATISTICAL ANALYSIS

Primary aims:

Since our primary aims are descriptive, they will be presented as frequencies, percentages, means and standard deviations as appropriate. Missing data will be calculated as the percentage amount of data available from the total amount of expected data. Engagement will be assessed through data availability in passive data streams and data quality will be measured as the number of active tasks that are incomplete. Associations between engagement and clinical characteristics will be explored. Recordings of the

For peer review only

For peer review only - http://bmjopen.bmj.com/site/about/guidelines.xhtml

semi-structured interviews will be transcribed verbatim, checked for accuracy by a second researcher and analysed using a deductive approach to thematic analysis, with the iterative categorisation technique [40]. Where participant responses can be quantified, they will be presented as aggregated counts or percentages, otherwise, summaries of participant responses will be presented narratively. Sporadic reports of issues with the technology or study methodologies will be summarised.

Secondary aims:
Digital features that account for sleep, activity, sociability, cognition will be extracted from sensor data and correlated against scores on scales of depression, anxiety and functionality. Feature extraction will replicate the methods used by the RADAR-CNS consortium [23,41]. Regression or classification analyses will be carried out for clinical scores to see whether higher impairment is associated with behavioural features. Regression and classification approaches will also be used to determine whether clinical data predict subject attrition or missing data patterns.

We will carry out univariate and multivariate associations on digital features and clinical state, as well as within and between individual comparisons. In order to unearth digital profiles in the sample, individuals will be clustered together based on their response patterns using latent class analysis. This person-centred approach will unpick some of the heterogeneity in the sample and assumes there are underlying latent variables that underpin distinct symptom profiles [42], and has been used extensively in the construction of the subtypes of depression [43]. This model will aid in the description of longitudinal behavioural patterns in this sample.

To evaluate the prognostic value of digital features, we will use machine learning methods on the extracted aggregated features and clinical information, provided there is sufficient data points. A multivariate prediction model will be constructed, and different feature selection algorithms will be applied. Model performance will be evaluated through cross-validation, putting stress on sensitivity and specificity of relapse prediction model. We will also use dynamic structural equation modelling to evaluate the lagged associations across study timepoints.

Where data is missing at random and assuming it is not significantly high, multiple imputation methods will be carried out. If missing data is high, this may be incorporated into the model as a predictor, or otherwise used informatively.

Reporting standards
In the interest of open and reproducible science, we will follow basic transparency recommendations[13,44–46], including the reporting of basic demographic and clinical data, attrition and participation rates, missing data, evidence of the validity or reliability of the sensors and devices used. For each behavioural feature, a full definition and description of feature construction will be provided, with links to Github repositories and source code, where available. Definition and handling of missing data will be specified. In machine learning models, model selection strategy, performance metrics and parameter estimates in the model with confidence intervals, or nonparametric equivalents will be described in full.

PPI statement
This research was reviewed by a team with experience of mental health problems and their carers who have been specially trained to advise on research proposals and documentation through the Feasibility and Acceptability Support Team for Researchers (FAST-R): a free, confidential service in England provided by the National Institute for Health Research Maudsley Biomedical Research Centre via King’s College London and South London and Maudsley NHS Foundation Trust.

ETHICS AND DISSEMINATION
This study has been reviewed and given favourable opinion by the London Westminster Research Ethics Committee, approval from the Health Research Authority, IRAS project ID: 270918, and confirmation of capacity and capability to carry out research from the South London and Maudsley NHS Foundation Trust. The research will be carried out in accordance with the Helsinki Declaration and International Conference on Harmonisation-Good Clinical Practice Guidelines. Privacy and confidentiality will be guaranteed and the procedures for handling, processing, storage and destruction of the data will comply with the General Data Protection Regulation (GDPR). Data collected will be hosted on KCL infrastructure. Participant Fitbit accounts will be created using generic email accounts so no personal details are shared with Fitbit.

The results of the study will be presented at local, national and international meetings or academic conferences, and will generate manuscripts to be submitted to peer-reviewed journals. Additionally, the results from this study will form part of a doctoral thesis and will be shared with participants, if they wish, after the study has been completed.

For peer review only - http://bmjopen.bmj.com/site/about/guidelines.xhtml

DISCUSSION

If digital technologies are to fulfil their potential to revolutionise the clinical management of mental health conditions, we need to establish the feasibility and acceptability of using RMTs such as wearables and smartphones to track mood and behaviour in those seeking and undergoing treatment for such conditions.

There are some anticipated challenges faced by this study. Continuous tracking of behaviours like physical activity and sleep may result in favourable changes to health behaviours and improved self-management. While the current study is non-interventional and does not aim to affect improvement rates, such behavioural changes may directly impact mood and treatment outcome.

The main concern, however, arises from the impact of the Covid-19 pandemic. Although the current public health crisis will impact this study in several ways, we identify three main areas. Firstly, part of the data collection will cover periods of time when there were government-imposed restrictions to movement and social proximity, meaning people's daily routine will have been greatly disrupted, and signals will bear additional noise. Secondly, the impact on individuals' mental health will be sizeable [47]. The profile of patients referred to psychological services may be different than before or after the pandemic, as we are faced with new mental health challenges [48]. Finally, the pandemic has resulted in the forced adoption of digital technology for all aspects of life, including healthcare, likely affecting attitudes towards technology and therefore engaging with and accepting RMTs [49].

Given the recency of the field and the interest in implementing digital technologies within healthcare, assessing the acceptability and feasibility of such methods in this target population is of great importance in informing implementation efforts as well as planning future research studies involving such samples. Through the use of mixed methods, the current study aims to identify and address as many of these issues as possible.

Funding:
This study represents independent research funded by the National Institute for Health Research (NIHR) Biomedical Research Centre at South London and Maudsley NHS Foundation Trust and King's College London. The views expressed are those of the authors and not necessarily those of the NHS, the NIHR or the Department of Health.

Acknowledgments:

RJBD is supported by the following: (1) NIHR Biomedical Research Centre at South London and Maudsley NHS Foundation Trust and King's College London, London, UK; (2) Health Data Research UK, which is funded by the UK Medical Research Council, Engineering and Physical Sciences Research Council, Economic and Social Research Council, Department of Health and Social Care (England), Chief Scientist Office of the Scottish Government Health and Social Care Directorates, Health and Social Care Research and Development Division (Welsh Government), Public Health Agency (Northern Ireland), British Heart Foundation and Wellcome Trust; (3) The BigData@Heart Consortium, funded by the Innovative Medicines Initiative-2 Joint Undertaking under grant agreement No. 116074. This Joint Undertaking receives support from the European Union's Horizon 2020 research and innovation programme and EFPIA; it is chaired by DE Grobbee and SD Anker, partnering with 20 academic and industry partners and ESC; (4) the National Institute for Health Research University College London Hospitals Biomedical Research Centre; (5) the National Institute for Health Research (NIHR) Biomedical Research Centre at South London and Maudsley NHS Foundation Trust and King's College London; (6) the UK Research and Innovation London Medical Imaging & Artificial Intelligence Centre for Value Based Healthcare; (7) the National Institute for Health Research (NIHR) Applied Research Collaboration South London (NIHR ARC South London) at King's College Hospital NHS Foundation Trust.

Competing Interests:
M.H. is principal investigator of the RADAR-CNS programme, a precompetitive public–private partnership funded by the Innovative Medicines Initiative and European Federation of Pharmaceutical Industries and Associations. The programme receives support from Janssen, Biogen, MSD, UCB and Lundbeck.

All other authors declare that they have no competing interests.

Authors' Contributions:

For peer review only - http://bmjopen.bmj.com/site/about/guidelines.xhtml

VdA, MH, RD and FM conceived and developed this project. SM and SL contributed to the design and implementation work within health services, as well as the acquisition of data. All authors contributed to the revision and edition of the manuscript and have provided their final approval of the current version.

List of Figures:

Figure 1. Study timeline for participants.
Figure 2. Participant flow in the RAPID study.

For peer review only

For peer review only - http://bmjopen.bmj.com/site/about/guidelines.xhtml

References:

1. World Health Organisation. Depression. https://www.who.int/news-room/fact-sheets/detail/depression (2020).
2. Cuijpers, P., Stringaris, A. & Wolpert, M. Treatment outcomes for depression: challenges and opportunities. *Lancet Psychiatry* **7**, 925–927 (2020).
3. Psychological Therapies, Annual report on the use of IAPT services - England, 2017-18 [PAS]. *NHS Digital* https://digital.nhs.uk/data-and-information/publications/statistical/psychological-therapies-annual-reports-on-the-use-of-iapt-services/annual-report-2017---18.
4. Wells, J. E. & Horwood, L. J. How accurate is recall of key symptoms of depression? A comparison of recall and longitudinal reports. *Psychol. Med.* **34**, 1001–1011 (2004).
5. Bilderbeck, A. C., Saunders, K. E. A., Price, J. & Goodwin, G. M. Psychiatric assessment of mood instability: qualitative study of patient experience. *Br. J. Psychiatry J. Ment. Sci.* **204**, 234–239 (2014).
6. Glenn, T. & Monteith, S. New Measures of Mental State and Behavior Based on Data Collected From Sensors, Smartphones, and the Internet. *Curr. Psychiatry Rep.* **16**, 523 (2014).
7. Lewis, G. *et al.* Variation in the recall of socially rewarding information and depressive symptom severity: a prospective cohort study. *Acta Psychiatr. Scand.* **135**, 489–498 (2017).
8. Mendlewicz, J. Sleep disturbances: Core symptoms of major depressive disorder rather than associated or comorbid disorders. *World J. Biol. Psychiatry* **10**, 269–275 (2009).
9. Cruwys, T. *et al.* Social group memberships protect against future depression, alleviate depression symptoms and prevent depression relapse. *Soc. Sci. Med.* **98**, 179–186 (2013).
10. Renn, B. N., Pratap, A., Atkins, D. C., Mooney, S. D. & Areán, P. A. Smartphone-Based Passive Assessment of Mobility in Depression: Challenges and Opportunities. *Ment. Health Phys. Act.* **14**, 136–139 (2018).
11. Cummins, N. *et al.* Generalized Two-Stage Rank Regression Framework for Depression Score Prediction from Speech. *IEEE Trans. Affect. Comput.* **11**, 272–283 (2020).
12. Simblett, S. *et al.* Barriers to and Facilitators of Engagement With Remote Measurement Technology for Managing Health: Systematic Review and Content Analysis of Findings. *J. Med. Internet Res.* **20**, e10480 (2018).
13. Angel, V. de *et al. Digital Health Tools for the Passive Monitoring of Depression: A Systematic Review of Methods*. 2021.07.19.21260786 https://www.medrxiv.org/content/10.1101/2021.07.19.21260786v1 (2021) doi:10.1101/2021.07.19.21260786.

For peer review only

For peer review only - http://bmjopen.bmj.com/site/about/guidelines.xhtml

14. Billingham, S. A., Whitehead, A. L. & Julious, S. A. An audit of sample sizes for pilot and feasibility trials being undertaken in the United Kingdom registered in the United Kingdom Clinical Research Network database. *BMC Med. Res. Methodol.* **13**, 104 (2013).

15. Sim, J. & Lewis, M. The size of a pilot study for a clinical trial should be calculated in relation to considerations of precision and efficiency. *J. Clin. Epidemiol.* **65**, 301–308 (2012).

16. Averill, I. R. *et al.* Clinical response to treatment in inpatients with depression correlates with changes in activity levels and psychomotor speed. *Aust. N. Z. J. Psychiatry* **52**, 652–659 (2018).

17. Berke, E. M., Choudhury, T., Ali, S. & Rabbi, M. Objective Measurement of Sociability and Activity: Mobile Sensing in the Community. *Ann. Fam. Med.* **9**, 344–350 (2011).

18. Szklo-Coxe, M., Young, T., Peppard, P. E., Finn, L. A. & Benca, R. M. Prospective Associations of Insomnia Markers and Symptoms With Depression. *Am. J. Epidemiol.* **171**, 709–720 (2010).

19. Sheehan, D. V. *et al.* The Mini-International Neuropsychiatric Interview (M.I.N.I.): the development and validation of a structured diagnostic psychiatric interview for DSM-IV and ICD-10. *J. Clin. Psychiatry* **59 Suppl 20**, 22-33;quiz 34-57 (1998).

20. Faurholt-Jepsen, M. *et al.* Differences in psychomotor activity in patients suffering from unipolar and bipolar affective disorder in the remitted or mild/moderate depressive state. *J. Affect. Disord.* **141**, 457–463 (2012).

21. Berle, J. O., Hauge, E. R., Oedegaard, K. J., Holsten, F. & Fasmer, O. B. Actigraphic registration of motor activity reveals a more structured behavioural pattern in schizophrenia than in major depression. *BMC Res. Notes* **3**, 149 (2010).

22. Stewart, J. W., McGrath, P. J., Rabkin, J. G. & Quitkin, F. M. Atypical Depression: A Valid Clinical Entity? *Psychiatr. Clin. North Am.* **16**, 479–495 (1993).

23. Ranjan, Y. *et al.* RADAR-base: A Novel Open Source m-Health Platform. in *Proceedings of the 2018 ACM International Joint Conference and 2018 International Symposium on Pervasive and Ubiquitous Computing and Wearable Computers - UbiComp '18* 223–226 (ACM Press, 2018). doi:10.1145/3267305.3267579.

24. Harris, P. A. *et al.* Research electronic data capture (REDCap)--a metadata-driven methodology and workflow process for providing translational research informatics support. *J. Biomed. Inform.* **42**, 377–381 (2009).

25. McIntyre, R. S. *et al.* The THINC-Integrated Tool (THINC-it) Screening Assessment for Cognitive Dysfunction: Validation in Patients With Major Depressive Disorder. *J. Clin. Psychiatry* **78**, 873–881 (2017).

26. Lam, R. W. *et al.* Psychometric validation of the Perceived Deficits Questionnaire-Depression (PDQ-D) instrument in US and UK respondents with major depressive disorder. *Neuropsychiatr. Dis. Treat.* **14**, 2861–2877 (2018).

27. Kroenke, K., Spitzer, R. L. & Williams, J. B. W. The PHQ-9. *J. Gen. Intern. Med.* **16**, 606–613 (2001).

For peer review only

For peer review only - http://bmjopen.bmj.com/site/about/guidelines.xhtml

28. Spitzer, R. L., Kroenke, K., Williams, J. B. W. & Löwe, B. A Brief Measure for Assessing Generalized Anxiety Disorder: The GAD-7. *Arch. Intern. Med.* **166**, 1092–1097 (2006).

29. Rush, A. J. *et al.* The 16-Item quick inventory of depressive symptomatology (QIDS), clinician rating (QIDS-C), and self-report (QIDS-SR): a psychometric evaluation in patients with chronic major depression. *Biol. Psychiatry* **54**, 573–583 (2003).

30. Mundt, J. C., Marks, I. M., Shear, M. K. & Greist, J. M. The Work and Social Adjustment Scale: a simple measure of impairment in functioning. *Br. J. Psychiatry* **180**, 461–464 (2002).

31. Hesse, M. & Moran, P. Screening for personality disorder with the Standardised Assessment of Personality: Abbreviated Scale (SAPAS): further evidence of concurrent validity. *BMC Psychiatry* **10**, 10 (2010).

32. Broadbent, E., Petrie, K. J., Main, J. & Weinman, J. The Brief Illness Perception Questionnaire. *J. Psychosom. Res.* **60**, 631–637 (2006).

33. Holmes, T. H. & Rahe, R. H. The social readjustment rating scale. *J. Psychosom. Res.* **11**, 213–218 (1967).

34. Bernstein, D. P. *et al.* Development and validation of a brief screening version of the Childhood Trauma Questionnaire. *Child Abuse Negl.* **27**, 169–190 (2003).

35. Bush, K., Kivlahan, D. R., McDonell, M. B., Fihn, S. D. & Bradley, K. A. The AUDIT Alcohol Consumption Questions (AUDIT-C): An Effective Brief Screening Test for Problem Drinking. *Arch. Intern. Med.* **158**, 1789–1795 (1998).

36. Dalgard, O. S. *et al.* Negative life events, social support and gender difference in depression: a multinational community survey with data from the ODIN study. *Soc. Psychiatry Psychiatr. Epidemiol.* **41**, 444–451 (2006).

37. Cohen, S., Kamarck, T. & Mermelstein, R. A Global Measure of Perceived Stress. *J. Health Soc. Behav.* **24**, 385–396 (1983).

38. Tracey, T. J. & Kokotovic, A. M. Factor structure of the Working Alliance Inventory. *Psychol. Assess. J. Consult. Clin. Psychol.* **1**, 207–210 (1989).

39. Treynor, W., Gonzalez, R. & Nolen-Hoeksema, S. Rumination Reconsidered: A Psychometric Analysis. *Cogn. Ther. Res.* **27**, 247–259 (2003).

40. Neale, J. Iterative categorization (IC): a systematic technique for analysing qualitative data. *Addiction* **111**, 1096–1106 (2016).

41. Zhang, Y. *et al.* Relationship Between Major Depression Symptom Severity and Sleep Collected Using a Wristband Wearable Device: Multicenter Longitudinal Observational Study. *JMIR MHealth UHealth* **9**, e24604 (2021).

42. Masyn, K. E. Latent Class Analysis and Finite Mixture Modeling. *Oxf. Handb. Quant. Methods Psychol. Vol 2* (2013) doi:10.1093/oxfordhb/9780199934898.013.0025.

43. Lanza, S. T. & Rhoades, B. L. Latent Class Analysis: An Alternative Perspective on Subgroup Analysis in Prevention and Treatment. *Prev. Sci.* **14**, 157–168 (2013).

For peer review only

For peer review only - http://bmjopen.bmj.com/site/about/guidelines.xhtml

44. Faurholt-Jepsen, M. *et al.* Reporting guidelines on remotely collected electronic mood data in mood disorder (eMOOD)—recommendations. *Transl. Psychiatry* **9**, 1–10 (2019).

45. Elm, E. von *et al.* The Strengthening the Reporting of Observational Studies in Epidemiology (STROBE) statement: guidelines for reporting observational studies. *The Lancet* **370**, 1453–1457 (2007).

46. Collins, G. S., Reitsma, J. B., Altman, D. G. & Moons, K. G. M. Transparent reporting of a multivariable prediction model for individual prognosis or diagnosis (TRIPOD): the TRIPOD statement. *BMJ* **350**, (2015).

47. Xiong, J. *et al.* Impact of COVID-19 pandemic on mental health in the general population: A systematic review. *J. Affect. Disord.* **277**, 55–64 (2020).

48. Tromans, S. *et al.* Patterns of use of secondary mental health services before and during COVID-19 lockdown: observational study. *BJPsych Open* **6**, (2020).

49. Hutchings, R. The impact of Covid-19 on the use of digital technology in the NHS. 23.

For peer review only

For peer review only - http://bmjopen.bmj.com/site/about/guidelines.xhtml

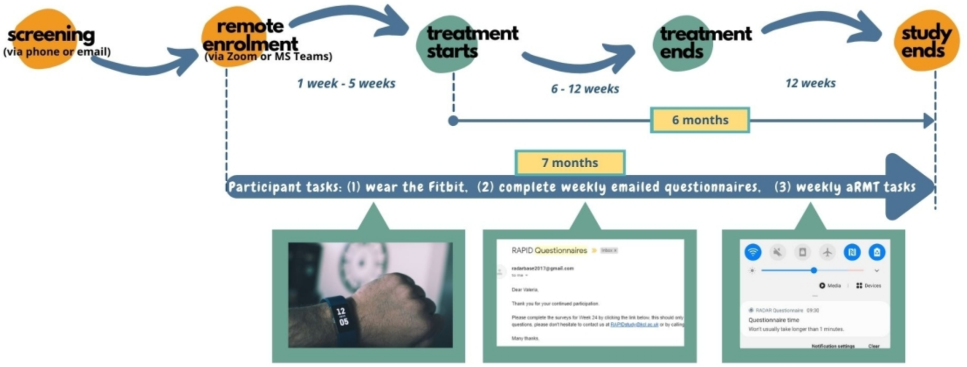

Figure 1. Study timeline for participants.

508x285mm (96 x 96 DPI)

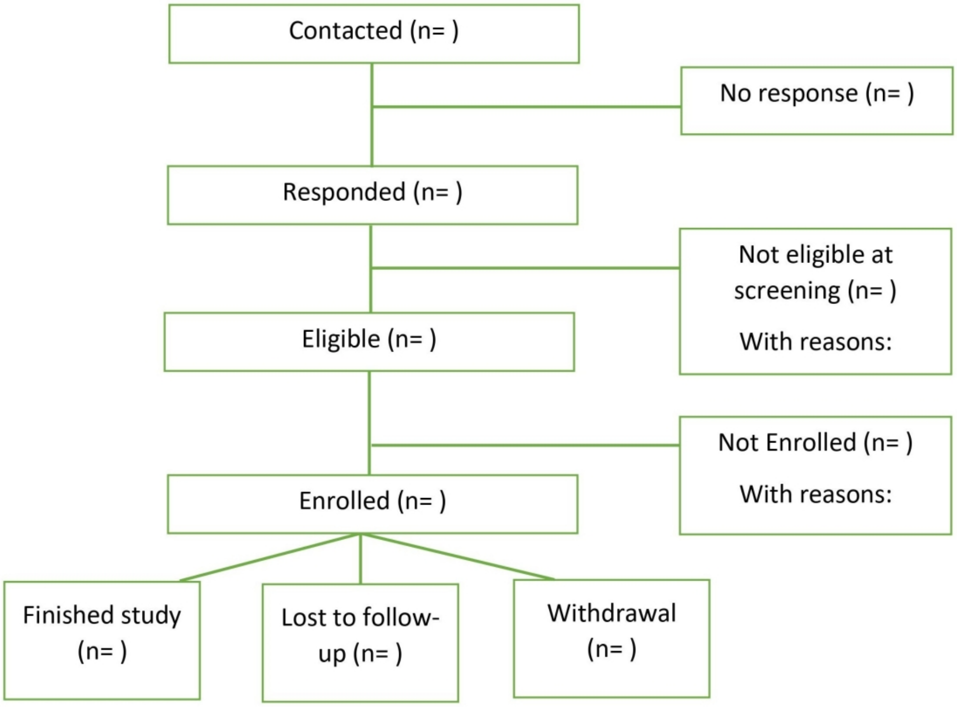

Figure 2. Participant flow in the RAPID study.

181x138mm (300 x 300 DPI)

BMJ Open

Using Digital Health Tools for the Remote Assessment of Treatment Prognosis In Depression (RAPID): A Study Protocol for a feasibility study

Journal:	*BMJ Open*
Manuscript ID	bmjopen-2021-059258.R1
Article Type:	Protocol
Date Submitted by the Author:	03-Mar-2022
Complete List of Authors:	de Angel, Valeria; King's College London, Department of Psychological Medicine<br>Lewis, Serena; King's College London; University of Bath, Department of Psychology<br>Munir, Sara; South London and Maudsley NHS Foundation Trust<br>Matcham, Faith; King's College London, Department of Psychological Medicine, IoPPN<br>Dobson, Richard; King's College London, Department of Biostatistics and Health Informatics; South London and Maudsley NHS Foundation Trust, NIHR Maudsley Biomedical Research Centre<br>Hotopf, Matthew; Institute of Psychiatry Psychology and Neuroscience Division of Academic Psychiatry
**Primary Subject Heading**:	Mental health
Secondary Subject Heading:	Epidemiology, Health informatics
Keywords:	Depression & mood disorders < PSYCHIATRY, Anxiety disorders < PSYCHIATRY, MENTAL HEALTH, Health informatics < BIOTECHNOLOGY & BIOINFORMATICS

SCHOLARONE™
Manuscripts

Using Digital Health Tools for the Remote Assessment of Treatment Prognosis In Depression (RAPID): A Study Protocol for a feasibility study

Valeria de Angel[1,2], Serena Lewis [1,3], Sara Munir [4], Faith Matcham [1], Richard Dobson [2,5], Matthew Hotopf [1,2]*

1. Institute of Psychiatry, Psychology and Neuroscience, King's College London, London, UK
2. NIHR Maudsley Biomedical Research Centre, South London and Maudsley NHS Foundation Trust, London, UK
3. Department of Psychology, University of Bath, Bath, UK
4. South London and Maudsley NHS Foundation Trust, London, UK
5. Department of Biostatistics and Health Informatics, Institute of Psychiatry, Psychology and Neuroscience (IoPPN), King's College London, 16 De Crespigny Park, London, SE5 8AF, UK

* Corresponding Author contact details:
E3.04, 3rd floor East Wing, Institute of Psychiatry, Psychology and Neuroscience
16 De Crespigny Park, London, SE5 8AF
E: Valeria.de_angel@kcl.ac.uk

Word count: 4062

Key words: Depression, Psychological therapy, Feasibility, Remote measurement technology, Passive sensing, mhealth, eMental health, digital phenotype, digital health tools, prospective cohort

For peer review only

For peer review only - http://bmjopen.bmj.com/site/about/guidelines.xhtml

Abstract:

Introduction:

Digital health tools such as smartphones and wearable devices could improve psychological treatment outcomes in depression through more accurate and comprehensive measures of patient behaviour. However, in this emerging field, most studies are small and based on student populations outside of a clinical setting. The current study aims to determine the feasibility and acceptability of using smartphones and wearable devices to collect behavioural and clinical data in people undergoing therapy for depressive disorders and establish the extent to which they can be potentially useful biomarkers of depression and recovery after treatment.

Methods and analysis:

This is an observational, prospective cohort study of 65 people attending psychological therapy for depression in multiple London-based sites. It will collect continuous passive data from smartphone sensors and a Fitbit fitness tracker, and deliver questionnaires, speech tasks and cognitive assessments through smartphone-based apps. Objective data on sleep, physical activity, location, Bluetooth contact, smartphone use, and heart rate, will be gathered for 7 months, and compared to clinical and contextual data. A mixed methods design, including a qualitative interview of patient experiences, will be used to evaluate key feasibility indicators, digital phenotypes of depression, and therapy prognosis. Public and Patient Involvement was sought for participant-facing documents and the study design of the current research proposal.

Ethics and dissemination.

Ethical approval has been obtained from the London Westminster Research Ethics Committee, and the Health Research Authority, IRAS project ID: 270918. Privacy and confidentiality will be guaranteed and the procedures for handling, processing, storage and destruction of the data will comply with the General Data Protection Regulation (GDPR). Findings from this study will form part of a doctoral thesis, will be presented at national and international meetings or academic conferences, and will generate manuscripts to be submitted to peer-reviewed journals.

Registration details: https://doi.org/10.17605/OSF.IO/PMYTA

Strengths and limitations of this study:

- The current mixed-methods design to evaluate feasibility and acceptability will provide a deeper understanding of the associations between patterns of missing data across the different data collection methods and clinical state.
- Both passive sensing and active validated questionnaire-based data collection methods will be evaluated.
- A 7-month participant follow-up provides a picture of engagement in the longer-term, compared to previous studies.
- For pragmatic reasons, the study uses a non-randomised, non-controlled design, which will limit conclusions about digital changes to treatment response.
- This study does not use devices that are validated for medical use, drawing instead from digital sensors in Android smartphones and a Fitbit fitness tracker, which have been previously used in mental health research.

For peer review only - http://bmjopen.bmj.com/site/about/guidelines.xhtml

INTRODUCTION

Depression is a leading cause of disability worldwide [1], yet response to treatment is poor, with only 50-60% of people recovering after 3 months of treatment [2,3]. Mental health science relies almost exclusively upon subjective self-report to diagnose mental illness and measure outcomes. Reliance is therefore placed upon patients being able to accurately recall and communicate complex mood states during clinical interviews, which many patients find difficult [4,5]. Depending on subjective methods introduces a vulnerability to recall biases [6] which may worsen with increased severity [7].

The use of digital tools within mental health has the potential to enhance traditional self-report measures by improving aspects of symptom tracking, illness management and treatment support. Remote measurement technologies (RMTs) such as smartphones and wearable devices can unobtrusively capture a more accurate picture of a patient's clinical state in a continuous way, with far less burden to the user. Through embedded sensors, they can detect changes in behaviours associated with depressive symptomatology such as sleep [8], sociability [9], physical activity [10], and speech [11]. Detecting such changes in a person's behaviour can provide invaluable information for tailoring and improving treatment.

Given the relative recency of the field, and with an eye towards clinical implementation, studies on RMTs and depression have largely comprised proof-of-concept, feasibility, and acceptability studies. While studies show RMTs to be generally feasible and acceptable [12], the data predominantly come from small, non-clinical or student samples with a median follow- up time of two weeks[13].

To our knowledge, no studies have been published on the use of RMTs to track mood and behaviour in clinical populations undergoing psychotherapy for depression. This population could derive greater benefits from the application of such digital methods by alleviating distress in more potentially severe cases and reducing pressure on healthcare services. In addition to the barriers of adopting RMTs for mood monitoring found across populations, such as concerns around privacy, confidentiality, affordability and accessibility, there are likely to be additional considerations related to the help-seeking populations that remain unexplored and may help or hinder implementation practices in the future.

From a clinical perspective, exploring feasibility and acceptability in therapy populations can shed light on how changes in severity affect engagement with and use of digital devices, how they can be used to complement treatment, and the barriers and facilitators to their use and implementation within services. From a methodological perspective, it would be important to establish the extent to which the amount and quality of data is usable and unbiased given that the added workload from therapy exercises, homework and clinical questionnaires as well as the severity of symptoms such as decreased motivation and cognitive abilities, are likely to affect engagement and device use [12]. Such studies could also inform future projects about the likely uptake in these samples in order to establish sample sizes and allocate resources.

Study aims:
The primary aim of this project is to evaluate the extent to which data collection with digital tools on clinical samples is feasible. Specifically, the feasibility of using RMTs, such as Android smartphones and a Fitbit wearable device, to collect behavioural and clinical data in people undergoing therapy for depressive disorders, to establish the extent to which they can be potentially useful biomarkers of depression and changes in clinical state. The purpose of this is to describe patterns of user engagement and missing data, which are likely to impact the scientific integrity of future large-scale studies.

Secondary aims are to identify candidate signals for digital biomarkers by detecting correlations between objective features and clinical characteristics, and to explore whether these signals have prognostic value in the context of psychological treatments.

METHODS AND ANALYSIS

Design
This is an observational, prospective cohort study of people attending psychological therapy for depression. It will use RMTs to gather active and passive data for up to 7 months and will adopt a mixed methods design to evaluate the feasibility and acceptability of such data collection methods.

Setting
Participants will be drawn from Improving Access to Psychological Therapies (IAPT) services in South London. The participating IAPT services will be from the boroughs of Lambeth, Lewisham and Croydon within the South London and Maudsley NHS

For peer review only - http://bmjopen.bmj.com/site/about/guidelines.xhtml

Foundation Trust. IAPT is a publicly funded self-referral outpatient programme providing evidence-based psychological treatments for adults with mild-to- moderate mental health disorders. The service is free at the point of delivery.

Recruitment for this study initiated in June 2020, during the COVID-19 pandemic. Given the government-imposed travel restrictions and social distancing measures, the entirety of this study will be carried out remotely.

Sample size

Formal sample size calculations are not a requirement for feasibility studies [14], however, the general recommendation is for samples of 50-60 participants to assess feasibility outcomes [15]. In order to address our secondary aims, a sample size of 50 would be sufficiently powered to detect a correlation coefficient of 0.39 and above, assuming a significance level of 0.05 and Type 2 error value of 0.20. Based on previous studies, we expect such an effect size [16–18]. To account for a potential attrition rate of 20% we will aim to recruit 65 participants.

Recruitment

IAPT clinicians will act as gatekeepers to the initial recruitment process. Patients within their service, who have previously agreed to be contacted for research purposes, will be invited to take part, either by phone call or email. They will be given a summary of the aims and procedures of the study and be screened for eligibility as per the inclusion/exclusion criteria below. When screening is done by email, participants receive a personalised email with a description of the study and a link to an online screening tool that participants can complete in their own time, the responses of which are relayed to the research team. If willing and eligible, potential participants will be sent the participant information sheet and given at least 24 hours before going through the consent procedures and being enrolled in the study.

Inclusion criteria:

a) Adults with a current depressive episode as measured by the Mini International Neuropsychiatric Interview (MINI [19]).
b) Being on the waiting list to receive treatment for depression at IAPT services, with an expected wait of at least 7 days (to a maximum of 5 weeks) between scheduled enrolment and first treatment session. Due to the prevalent comorbidity with anxiety disorders, people with a main diagnosis of anxiety were also included, provided they met inclusion criteria a).
c) Existing ownership of Android smartphone with sufficient memory space for the relevant apps.
d) Able and willing to use a wrist worn device for duration of the study
e) Able to give informed consent for participation.
f) Sufficient English language skills to understand consent process and questionnaires.

Exclusion criteria:

a) Lifetime diagnosis of bipolar disorder, schizophrenia and schizoaffective disorders as these have different digital patterns to depression [20,21]
b) Health anxieties that may significantly worsen with constant monitoring of behaviour.
c) Extensive sharing of smartphone with friends or family
d) Night shifts, pregnancy, or living with a 0–6-month-old baby (due to sleep disruptions)

Study procedures

Once interest and eligibility have been ascertained, participants will be invited to attend an enrolment session via videocall. Figure 1 shows the study timeline for participants as they enter the study. After a further review of study procedures and opportunity for questions, participants will be asked to sign an electronic consent form. Consent can be taken either using the Qualtrics platform, which has been approved for this purpose, or via MS Word or PDF documents which participants electronically sign from their devices.

Enrolment/Baseline

Following consent, the enrolment session is comprised of three further sections: (1) obtaining sociodemographic and clinical data, (2) completion of self-reported questionnaires, and (3) technology set up. The researchers will take demographic and clinical information related to current and previous physical and mental health conditions, family history, treatment status as well as phone use, previous experience with health apps and devices, social and physical activity levels. In order to detect the presence of a depressive episode, and define whether atypical in nature, MINI and the Atypical Depression Diagnostic Scale (ADDS) [22] will be administered. At the end of the session, participants will be asked to complete a battery of self-reported questionnaires as shown in Table 1.

Technology set-up

For peer review only - http://bmjopen.bmj.com/site/about/guidelines.xhtml
For peer review only

Participants will be asked to download 4 apps on their phone; RADAR passive RMT (pRMT) app, which collects background sensor data from smartphones; the RADAR active RMT (aRMT) app, which delivers clinical questionnaires; THINC-it® for RADAR-CNS, an app assessing cognitive function; and the Fitbit app. These will be linked in-call to the RADAR-base platform [23]. A Fitbit Charge 3 or 4 is then delivered to them within 1-2 working days, at which point they are guided through the set up. Participants will be given £10.00 for completing the enrolment session and keep the Fitbit after the study. Table 1 shows the schedule of events for the RAPID study.

Follow-up:
From enrolment, longitudinal collection of active and passive data begins. The current study will use the RADAR-base platform and their apps to collect passive and active data, as well as a Fitbit API integration source [23]. More information can be found at radar-base.org/.

Passive Measures:
Passive measures will be continuously gathered from smartphone sensors via the pRMT app and wearable sensors from the Fitbit. Sensor data will include GPS, acceleration, light, phone interaction (total time on phone and app usage), paired and nearby Bluetooth devices, number of saved contacts, battery level and weather. Fitbit generates digital features relating to sleep, physical activity and heart rate.

Neither the Fitbit nor the apps are validated medical tools, as they are not intended to diagnose or treat a medical condition; RADAR-based apps are purpose-built for research, while the Fitbit is marketed as a fitness tracker. Despite questions surrounding the ability of digital sensors in detecting the behaviours of interest accurately, they have been found to reliably detect sleep, physical activity and location [24–26]

No personally identifiable information will be gathered from these sensors; GPS signals are obfuscated and relative to previous location rather than exact points, and no contact details, website or app content is collected by the apps. Personal privacy is thus protected, and no identification of an individual's home address or precise geographical location can be gathered.

Active measures:
Participants will be asked to respond to questionnaires throughout the study period. Some of them will be delivered via the aRMT app, others will be collected via the Research Electronic Data Capture (REDCap) software [27], a web-based platform for research that sends e-mail notifications to participants throughout the study.

(a) Weekly emailed questionnaires: Participants will receive weekly emails with a link to complete REDCap-delivered questionnaires, which can be complete on a smartphones or a computer. Questionnaires will be scheduled at different time intervals (fortnightly, monthly), in such a way that the maximum amount of time needed to complete them is 10 minutes per week.
(b) Weekly aRMT tasks: The aRMT app is designed to collect health information from research participants by sending them notifications and asking them to complete in-app tasks and questionnaires. These will include questions on depression, functionality, subjective sleep experiences and a speech task.
(c) Speech Task: Participants will be asked to undertake 2 speech tasks. The first task will require them to read out pre-written text, and the second task will ask them to answer out loud a question such as: "Can you describe something you are looking forward to this week?". Participants will record their voice in quiet surroundings for both tasks via the aRMT app. Acoustic features such as pitch, jitter, shimmer, formants and intensity will be extracted.
(d) Cognition via THINC-it® app: Once a month, the aRMT app will notify participants that it's time to complete the THINC-it tasks, they will be asked to open the THINC-it app to do so. THINC-it is a validated tool designed to assess cognitive function in depression [28]. The tests incorporated in this tool – the One-Back Test, the Trail Making Test Part B, the Digit Symbol Substitution Test, Choice Reaction Time task – assess attention, processing speed, executive function, learning and memory. The tool also incorporates the Perceived Deficits Questionnaire [29], a self-report questionnaire that assesses a person's cognitive concerns.

Table 1. Schedule of events.

Event	Enrolment	Questionnaire Frequency			
		Weekly	Fortnightly	Monthly	Endpoint
Informed Consent	x				
Sociodemographic data	x				

For peer review only - http://bmjopen.bmj.com/site/about/guidelines.xhtml

Clinical History	x				
MINI	x				
ADDS	x				
Smartphone apps set-up	x				
**Active Measures[a] (from REDCap)**					
Validated Questionnaires:					
SAPAS	x				
BIPQ	x				x
Life stress (SRRS)	x				x
CTQ	x				
AUDIT	x				
PHQ – 9	x		x		x
GAD-7	x		x		x
Rumination (RRS)	x			x	
AUDIT (short version)	x		x		
Oslo 3 Social support	x			x	
Perceived Stress Scale	x			x	x
WAI – SR**					x
Contextual information:					
Caffeine Intake	x	x			
Treatment status and content*	x	x			
Social activities	x		x		
Social distancing practices	x		x		
COVID experience	x			x	
**Active Measures[a] (from aRMT app):**					
(Q)IDS – SR	x	x			
WSAS	x	x			x
Speech Task	x		x		
Perceived sleep[ɪ]	x	x			
Cognition (THINC-it app)	x			x	
**Passive Measures:**					
Fitbit Charge	To be worn throughout the study.				
pRMT app: GPS, acceleration, light, phone interaction Bluetooth devices, number of contacts, battery level, weather,	Will run in the background gathering data from enrolment.				
**Qualitative Interview****					x

[a] Weekly time spent completing questionnaires should not exceed 10 mins.
* Only for the duration of treatment.
** Completed once during treatment
[ɪ] Daily for 90 days.

End of study:
Twelve weeks after participants have finished treatment, the research team will contact them to finalise their time in the study and complete endpoint assessments. In case of an unexpected change regarding their treatment, such as treatment being reduced or extended, the 3-month follow-up will commence on the day of their last core treatment session with IAPT services.

Extra Participant contact:
To maintain engagement and stay abreast of any issues, participants will be contacted after their first week post enrolment, and then in months 1, 3, 5 and 7. Researchers will initially contact participants on the phone unless an alternative method of contact is preferred and followed up with an email. Any issues raised in these calls, or sporadically reported by participants throughout the course of the

For peer review only

For peer review only - http://bmjopen.bmj.com/site/about/guidelines.xhtml

study will be recorded. Additionally, participants will be sent a monthly newsletter, via e-mail, which will include study updates, tech tips and any frequently asked questions.

Post-treatment Qualitative Interview:
In order to inform the feasibility and acceptability aims of this study, participants who complete therapy will be invited to take part in an optional qualitative interview. It will be a 30-minute semi-structured interview looking at participant experiences of using RMTs during psychotherapy for depression. We will invite participants to this interview once they complete treatment and will interview the first 20 who agree to take part. See supplementary materials for a full interview schedule.

Outcome measures

Primary outcomes

The primary outcome is to establish the feasibility of using wearable devices and smartphone sensors to monitor the behaviour of people with depression whilst receiving psychological treatment. Key feasibility outcomes will be related to clinical and methodological considerations, and will evaluate recruitment and participant flow, subjective reports of acceptability of methods, data availability and data quality.
The following feasibility outcomes will be reported:

(1) Estimates of recruitment and attrition rates (Figure 2)
(2) Presence and absence of passive data: 'wear time' for wearable devices and 'on time' for smartphone sensors, and the extent to which the available data allows for correlational and predictive analyses with significant statistical power.
(3) Active data availability and data quality: percentage number of tasks completed.
(4) Qualitative data: Participant experience and attitudes towards data collection instruments and procedures.

Secondary Outcomes

Secondary outcomes will evaluate the relationship between digital data and clinical outcome measures, both at individual time points and as prognostic factors for recovery after treatment. Scores on clinical scales will be used in addition to individual symptom domains.

Digital outcomes will be derived from smartphone and Fitbit sensors, and digital features from passive measures described in the "Passive Measures" section, such as GPS signal patterns, sleep, phone use and Bluetooth interactions. They will be extracted to form averages that encapsulate daily, weekly and within-treatment means and standard deviations or frequency counts.
)

Clinical state will be measured using:

- Patient Health Questionnaire (PHQ – 9) [30]: the 9-item questionnaire that is widely used for measuring depression in IAPT services.
- Generalised Anxiety Disorder questionnaire (GAD-7) [31]: will be measured as it gathers important anxiety symptoms which are so often comorbid with depression.
- Quick Inventory of Depressive Symptomatology – Self-rated (QIDS – SR)[32]: This is a 16-item inventory of depression for patients who identify as depressed or who may be suffering from depression.
- Work and Social Adjustment Scale (WSAS)[33]: a measure of quality-of-life/disability, which is a 5-item assessment of perceived social and work-related functional impairment used widely across a range of mental and physical disorders.
- Cognition via the THINC-it app.

Participants will be considered to be in remission if they have experienced a reduction of at least 50% in depressive symptomatology from the start of treatment, or no longer meet criteria for depression according to the PHQ-9 (scoring below the cut-off of 5). Sub-dimensions of depression – for example interest in activities, motivation, appetite – will be gathered from the QIDS-SR.

The socio-demographic, clinical and contextual variables measured, such as illness severity, cognitive function and social support will be taken as covariates.

- Standardised Assessment of Personality: Abbreviated Scale (SAPAS) [34] – an 8-item personality test that screens for personality disorder.
- Brief Illness Perceptions Questionnaire [35]. The BIPQ provides an insight into the participant's views about their underlying condition and how well they see themselves coping with it.
- Life stress scale (SRRS) [36] – this is a retrospective questionnaire for identifying major stressful life events.
- Childhood Trauma Questionnaire (CTQ) [37] – a 26-item scale that assesses five types of maltreatment; sexual abuse, physical abuse, emotional neglect, physical neglect and emotional abuse.

For peer review only - http://bmjopen.bmj.com/site/about/guidelines.xhtml
For peer review only

- Alcohol Use Disorder Identification Test (AUDIT) [38] – widely used scale in primary care that measures alcohol consumption, drinking behaviours and identifies harmful alcohol use.
- Oslo 3-items social support scale (OSSS – 3) [39] – is a brief instrument that assesses social support.
- Perceived Stress Scale (PSS) [40] – It is a measure of the degree to which situations in one's life are appraised as stressful. Items were designed to assess how unpredictable, uncontrollable, and overloaded respondents find their lives to be.
- Working Alliance Inventory – Short-Revised (WAI-SR) [41]: a 12-item scale measuring therapeutic alliance on 3 key components: (a) agreement on the tasks of therapy, (b) agreement on the goals of therapy and (c) development of an affective bond.
- Rumination Response Scale (RRS) [42] – a 10 item scale that measures rumination.
- COVID related questions – isolation status and their perceived effect, confirmed or suspected diagnosis of COVID19 as well as social distancing practices.
- Treatment status – questions on whether psychotherapy has begun, whether they take concomitant medication and adherence (e.g. "Are you taking medication for your mental health? If so, which one?" or "Have you missed any psychotherapy sessions this week?"), and the broad content of their therapy sessions.

STATISTICAL ANALYSIS

Primary aims:
Since our primary aims are descriptive, they will be presented as frequencies, percentages, means and standard deviations as appropriate. Missing data will be calculated as the percentage amount of data available from the total amount of expected data. Engagement will be assessed through data availability in passive data streams and data quality will be measured as the number of active tasks that are incomplete. Associations between engagement and clinical characteristics will be explored. Recordings of the semi-structured interviews will be transcribed verbatim, checked for accuracy by a second researcher and analysed using a deductive approach to thematic analysis, with the iterative categorisation technique [43]. Where participant responses can be quantified, they will be presented as aggregated counts or percentages, otherwise, summaries of participant responses will be presented narratively. Sporadic reports of issues with the technology or study methodologies will be summarised.

Secondary aims:
Digital features that account for sleep, activity, sociability, cognition will be extracted from sensor data and correlated against scores on scales of depression, anxiety and functionality. Feature extraction will replicate the methods used by the RADAR-CNS consortium [23,44]. Regression or classification analyses will be carried out for clinical scores to see whether higher impairment is associated with behavioural features. Regression and classification approaches will also be used to determine whether clinical data predict subject attrition or missing data patterns.

We will carry out univariate and multivariate associations on digital features and clinical state, as well as within and between individual comparisons. In order to unearth digital profiles in the sample, individuals will be clustered together based on their response patterns using latent class analysis. This person-centred approach will unpick some of the heterogeneity in the sample and assumes there are underlying latent variables that underpin distinct symptom profiles [45], and has been used extensively in the construction of the subtypes of depression [46]. This model will aid in the description of longitudinal behavioural patterns in this sample.

To evaluate the prognostic value of digital features, we will use machine learning methods on the extracted aggregated features and clinical information, provided there is sufficient data points. A multivariate prediction model will be constructed, and different feature selection algorithms will be applied. Model performance will be evaluated through cross-validation, putting stress on sensitivity and specificity of relapse prediction model. We will also use dynamic structural equation modelling to evaluate the lagged associations across study timepoints.

Where data is missing at random and assuming it is not significantly high, multiple imputation methods will be carried out. If missing data is high, this may be incorporated into the model as a predictor, or otherwise used informatively.

Reporting standards

In the interest of open and reproducible science, we will follow basic transparency recommendations[13,47–49], including the reporting of basic demographic and clinical data, attrition and participation rates, missing data, evidence of the validity or reliability of the sensors and devices used. For each behavioural feature, a full definition and description of feature construction will be provided, with links to Github repositories and source code, where available. Definition and handling of missing data will be specified. In

For peer review only - http://bmjopen.bmj.com/site/about/guidelines.xhtml
For peer review only

machine learning models, model selection strategy, performance metrics and parameter estimates in the model with confidence intervals, or nonparametric equivalents will be described in full.

PPI statement

This research was reviewed by a team with experience of mental health problems and their carers who have been specially trained to advise on research proposals and documentation through the Feasibility and Acceptability Support Team for Researchers (FAST-R): a free, confidential service in England provided by the National Institute for Health Research Maudsley Biomedical Research Centre via King's College London and South London and Maudsley NHS Foundation Trust.

ETHICS AND DISSEMINATION

This study has been reviewed and given favourable opinion by the London Westminster Research Ethics Committee, approval from the Health Research Authority, IRAS project ID: 270918, and confirmation of capacity and capability to carry out research from the South London and Maudsley NHS Foundation Trust. The research will be carried out in accordance with the Helsinki Declaration and International Conference on Harmonisation-Good Clinical Practice Guidelines. Privacy and confidentiality will be guaranteed and the procedures for handling, processing, storage and destruction of the data will comply with the General Data Protection Regulation (GDPR). Data collected will be hosted on KCL infrastructure. Participant Fitbit accounts will be created using generic email accounts so no personal details are shared with Fitbit.

The results of the study will be presented at local, national and international meetings or academic conferences, and will generate manuscripts to be submitted to peer-reviewed journals. Additionally, the results from this study will form part of a doctoral thesis and will be shared with participants, if they wish, after the study has been completed.

DISCUSSION

If digital technologies are to fulfil their potential to revolutionise the clinical management of mental health conditions, we need to establish the feasibility and acceptability of using RMTs such as wearables and smartphones to track mood and behaviour in those seeking and undergoing treatment for such conditions.

There are some anticipated challenges faced by this study. Continuous tracking of behaviours like physical activity and sleep may result in favourable changes to health behaviours and improved self-management. While the current study is non-interventional and does not aim to affect improvement rates, such behavioural changes may directly impact mood and treatment outcome.

The main concern, however, arises from the impact of the Covid-19 pandemic. Although the current public health crisis will impact this study in several ways, we identify three main areas. Firstly, part of the data collection will cover periods of time when there were government-imposed restrictions to movement and social proximity, meaning people's daily routine will have been greatly disrupted, and signals will bear additional noise. Secondly, the impact on individuals' mental health will be sizeable [50]. The profile of patients referred to psychological services may be different than before or after the pandemic, as we are faced with new mental health challenges [51]. Finally, the pandemic has resulted in the forced adoption of digital technology for all aspects of life, including healthcare, likely affecting attitudes towards technology and therefore engaging with and accepting RMTs [52].

Given the recency of the field and the interest in implementing digital technologies within healthcare, assessing the acceptability and feasibility of such methods in this target population is of great importance in informing implementation efforts as well as planning future research studies involving such samples. Through the use of mixed methods, the current study aims to identify and address as many of these issues as possible.

Funding:

This study represents independent research funded by the National Institute for Health Research (NIHR) Biomedical Research Centre at South London and Maudsley NHS Foundation Trust and King's College London. The views expressed are those of the authors and not necessarily those of the NHS, the NIHR or the Department of Health.

Acknowledgments:

For peer review only - http://bmjopen.bmj.com/site/about/guidelines.xhtml

RJBD is supported by the following: (1) NIHR Biomedical Research Centre at South London and Maudsley NHS Foundation Trust and King's College London, London, UK; (2) Health Data Research UK, which is funded by the UK Medical Research Council, Engineering and Physical Sciences Research Council, Economic and Social Research Council, Department of Health and Social Care (England), Chief Scientist Office of the Scottish Government Health and Social Care Directorates, Health and Social Care Research and Development Division (Welsh Government), Public Health Agency (Northern Ireland), British Heart Foundation and Wellcome Trust; (3) The BigData@Heart Consortium, funded by the Innovative Medicines Initiative-2 Joint Undertaking under grant agreement No. 116074. This Joint Undertaking receives support from the European Union's Horizon 2020 research and innovation programme and EFPIA; it is chaired by DE Grobbee and SD Anker, partnering with 20 academic and industry partners and ESC; (4) the National Institute for Health Research University College London Hospitals Biomedical Research Centre; (5) the National Institute for Health Research (NIHR) Biomedical Research Centre at South London and Maudsley NHS Foundation Trust and King's College London; (6) the UK Research and Innovation London Medical Imaging & Artificial Intelligence Centre for Value Based Healthcare; (7) the National Institute for Health Research (NIHR) Applied Research Collaboration South London (NIHR ARC South London) at King's College Hospital NHS Foundation Trust.

Competing Interests:
M.H. is principal investigator of the RADAR-CNS programme, a precompetitive public–private partnership funded by the Innovative Medicines Initiative and European Federation of Pharmaceutical Industries and Associations. The programme receives support from Janssen, Biogen, MSD, UCB and Lundbeck.

All other authors declare that they have no competing interests.

Authors' Contributions:
VdA, MH, RD and FM conceived and developed this project. SM and SL contributed to the design and implementation work within health services, as well as the acquisition of data. All authors contributed to the revision and edition of the manuscript and have provided their final approval of the current version.

List of Figures:
Figure 1. Study timeline for participants.
Figure 2. Participant flow in the RAPID study.

For peer review only

For peer review only - http://bmjopen.bmj.com/site/about/guidelines.xhtml

References:

1. World Health Organization. *Depression and other common mental disorders: global health estimates*. (2017).
2. Cuijpers, P., Stringaris, A. & Wolpert, M. Treatment outcomes for depression: challenges and opportunities. *Lancet Psychiatry* **7**, 925–927 (2020).
3. Psychological Therapies, Annual report on the use of IAPT services - England, 2017-18 [PAS]. *NHS Digital* https://digital.nhs.uk/data-and-information/publications/statistical/psychological-therapies-annual-reports-on-the-use-of-iapt-services/annual-report-2017---18.
4. Wells, J. E. & Horwood, L. J. How accurate is recall of key symptoms of depression? A comparison of recall and longitudinal reports. *Psychol. Med.* **34**, 1001–1011 (2004).
5. Bilderbeck, A. C., Saunders, K. E. A., Price, J. & Goodwin, G. M. Psychiatric assessment of mood instability: qualitative study of patient experience. *Br. J. Psychiatry J. Ment. Sci.* **204**, 234–239 (2014).
6. Glenn, T. & Monteith, S. New Measures of Mental State and Behavior Based on Data Collected From Sensors, Smartphones, and the Internet. *Curr. Psychiatry Rep.* **16**, 523 (2014).
7. Lewis, G. *et al.* Variation in the recall of socially rewarding information and depressive symptom severity: a prospective cohort study. *Acta Psychiatr. Scand.* **135**, 489–498 (2017).
8. Mendlewicz, J. Sleep disturbances: Core symptoms of major depressive disorder rather than associated or comorbid disorders. *World J. Biol. Psychiatry* **10**, 269–275 (2009).
9. Cruwys, T. *et al.* Social group memberships protect against future depression, alleviate depression symptoms and prevent depression relapse. *Soc. Sci. Med.* **98**, 179–186 (2013).
10. Renn, B. N., Pratap, A., Atkins, D. C., Mooney, S. D. & Areán, P. A. Smartphone-Based Passive Assessment of Mobility in Depression: Challenges and Opportunities. *Ment. Health Phys. Act.* **14**, 136–139 (2018).
11. Cummins, N. *et al.* Generalized Two-Stage Rank Regression Framework for Depression Score Prediction from Speech. *IEEE Trans. Affect. Comput.* **11**, 272–283 (2020).
12. Simblett, S. *et al.* Barriers to and Facilitators of Engagement With Remote Measurement Technology for Managing Health: Systematic Review and Content Analysis of Findings. *J. Med. Internet Res.* **20**, e10480 (2018).
13. De Angel, V. *et al.* Digital health tools for the passive monitoring of depression: a systematic review of methods. *Npj Digit. Med.* **5**, 1–14 (2022).
14. Billingham, S. A., Whitehead, A. L. & Julious, S. A. An audit of sample sizes for pilot and feasibility trials being undertaken in the United Kingdom registered in the United Kingdom Clinical Research Network database. *BMC Med. Res. Methodol.* **13**, 104 (2013).

For peer review only - http://bmjopen.bmj.com/site/about/guidelines.xhtml

15. Sim, J. & Lewis, M. The size of a pilot study for a clinical trial should be calculated in relation to considerations of precision and efficiency. *J. Clin. Epidemiol.* **65**, 301–308 (2012).

16. Averill, I. R. *et al.* Clinical response to treatment in inpatients with depression correlates with changes in activity levels and psychomotor speed. *Aust. N. Z. J. Psychiatry* **52**, 652–659 (2018).

17. Berke, E. M., Choudhury, T., Ali, S. & Rabbi, M. Objective Measurement of Sociability and Activity: Mobile Sensing in the Community. *Ann. Fam. Med.* **9**, 344–350 (2011).

18. Szklo-Coxe, M., Young, T., Peppard, P. E., Finn, L. A. & Benca, R. M. Prospective Associations of Insomnia Markers and Symptoms With Depression. *Am. J. Epidemiol.* **171**, 709–720 (2010).

19. Sheehan, D. V. *et al.* The Mini-International Neuropsychiatric Interview (M.I.N.I.): the development and validation of a structured diagnostic psychiatric interview for DSM-IV and ICD-10. *J. Clin. Psychiatry* **59 Suppl 20**, 22-33;quiz 34-57 (1998).

20. Faurholt-Jepsen, M. *et al.* Differences in psychomotor activity in patients suffering from unipolar and bipolar affective disorder in the remitted or mild/moderate depressive state. *J. Affect. Disord.* **141**, 457–463 (2012).

21. Berle, J. O., Hauge, E. R., Oedegaard, K. J., Holsten, F. & Fasmer, O. B. Actigraphic registration of motor activity reveals a more structured behavioural pattern in schizophrenia than in major depression. *BMC Res. Notes* **3**, 149 (2010).

22. Stewart, J. W., McGrath, P. J., Rabkin, J. G. & Quitkin, F. M. Atypical Depression: A Valid Clinical Entity? *Psychiatr. Clin. North Am.* **16**, 479–495 (1993).

23. Ranjan, Y. *et al.* RADAR-base: A Novel Open Source m-Health Platform. in *Proceedings of the 2018 ACM International Joint Conference and 2018 International Symposium on Pervasive and Ubiquitous Computing and Wearable Computers - UbiComp '18* 223–226 (ACM Press, 2018). doi:10.1145/3267305.3267579.

24. Adamakis, M. Comparing the Validity of a GPS Monitor and a Smartphone Application to Measure Physical Activity. *J. Mob. Technol. Med.* **6**, 28–38 (2017).

25. Plasqui, G., Bonomi, A. G. & Westerterp, K. R. Daily physical activity assessment with accelerometers: new insights and validation studies. *Obes. Rev.* **14**, 451–462 (2013).

26. Conley, S. *et al.* Agreement between actigraphic and polysomnographic measures of sleep in adults with and without chronic conditions: A systematic review and meta-analysis. *Sleep Med. Rev.* **46**, 151–160 (2019).

27. Harris, P. A. *et al.* Research electronic data capture (REDCap)--a metadata-driven methodology and workflow process for providing translational research informatics support. *J. Biomed. Inform.* **42**, 377–381 (2009).

28. McIntyre, R. S. *et al.* The THINC-Integrated Tool (THINC-it) Screening Assessment for Cognitive Dysfunction: Validation in Patients With Major Depressive Disorder. *J. Clin. Psychiatry* **78**, 873–881 (2017).

For peer review only

For peer review only - http://bmjopen.bmj.com/site/about/guidelines.xhtml

29. Lam, R. W. *et al.* Psychometric validation of the Perceived Deficits Questionnaire-Depression (PDQ-D) instrument in US and UK respondents with major depressive disorder. *Neuropsychiatr. Dis. Treat.* **14**, 2861–2877 (2018).

30. Kroenke, K., Spitzer, R. L. & Williams, J. B. W. The PHQ-9. *J. Gen. Intern. Med.* **16**, 606–613 (2001).

31. Spitzer, R. L., Kroenke, K., Williams, J. B. W. & Löwe, B. A Brief Measure for Assessing Generalized Anxiety Disorder: The GAD-7. *Arch. Intern. Med.* **166**, 1092–1097 (2006).

32. Rush, A. J. *et al.* The 16-Item quick inventory of depressive symptomatology (QIDS), clinician rating (QIDS-C), and self-report (QIDS-SR): a psychometric evaluation in patients with chronic major depression. *Biol. Psychiatry* **54**, 573–583 (2003).

33. Mundt, J. C., Marks, I. M., Shear, M. K. & Greist, J. M. The Work and Social Adjustment Scale: a simple measure of impairment in functioning. *Br. J. Psychiatry* **180**, 461–464 (2002).

34. Hesse, M. & Moran, P. Screening for personality disorder with the Standardised Assessment of Personality: Abbreviated Scale (SAPAS): further evidence of concurrent validity. *BMC Psychiatry* **10**, 10 (2010).

35. Broadbent, E., Petrie, K. J., Main, J. & Weinman, J. The Brief Illness Perception Questionnaire. *J. Psychosom. Res.* **60**, 631–637 (2006).

36. Holmes, T. H. & Rahe, R. H. The social readjustment rating scale. *J. Psychosom. Res.* **11**, 213–218 (1967).

37. Bernstein, D. P. *et al.* Development and validation of a brief screening version of the Childhood Trauma Questionnaire. *Child Abuse Negl.* **27**, 169–190 (2003).

38. Bush, K., Kivlahan, D. R., McDonell, M. B., Fihn, S. D. & Bradley, K. A. The AUDIT Alcohol Consumption Questions (AUDIT-C): An Effective Brief Screening Test for Problem Drinking. *Arch. Intern. Med.* **158**, 1789–1795 (1998).

39. Dalgard, O. S. *et al.* Negative life events, social support and gender difference in depression: a multinational community survey with data from the ODIN study. *Soc. Psychiatry Psychiatr. Epidemiol.* **41**, 444–451 (2006).

40. Cohen, S., Kamarck, T. & Mermelstein, R. A Global Measure of Perceived Stress. *J. Health Soc. Behav.* **24**, 385–396 (1983).

41. Tracey, T. J. & Kokotovic, A. M. Factor structure of the Working Alliance Inventory. *Psychol. Assess. J. Consult. Clin. Psychol.* **1**, 207–210 (1989).

42. Treynor, W., Gonzalez, R. & Nolen-Hoeksema, S. Rumination Reconsidered: A Psychometric Analysis. *Cogn. Ther. Res.* **27**, 247–259 (2003).

43. Neale, J. Iterative categorization (IC): a systematic technique for analysing qualitative data. *Addiction* **111**, 1096–1106 (2016).

44. Zhang, Y. *et al.* Relationship Between Major Depression Symptom Severity and Sleep Collected Using a Wristband Wearable Device: Multicenter Longitudinal Observational Study. *JMIR MHealth UHealth* **9**, e24604 (2021).

45. Masyn, K. E. Latent Class Analysis and Finite Mixture Modeling. *Oxf. Handb. Quant. Methods Psychol. Vol 2* (2013) doi:10.1093/oxfordhb/9780199934898.013.0025.

For peer review only

For peer review only - http://bmjopen.bmj.com/site/about/guidelines.xhtml

46. Lanza, S. T. & Rhoades, B. L. Latent Class Analysis: An Alternative Perspective on Subgroup Analysis in Prevention and Treatment. *Prev. Sci.* **14**, 157–168 (2013).

47. Faurholt-Jepsen, M. *et al.* Reporting guidelines on remotely collected electronic mood data in mood disorder (eMOOD)—recommendations. *Transl. Psychiatry* **9**, 1–10 (2019).

48. Elm, E. von *et al.* The Strengthening the Reporting of Observational Studies in Epidemiology (STROBE) statement: guidelines for reporting observational studies. *The Lancet* **370**, 1453–1457 (2007).

49. Collins, G. S., Reitsma, J. B., Altman, D. G. & Moons, K. G. M. Transparent reporting of a multivariable prediction model for individual prognosis or diagnosis (TRIPOD): the TRIPOD statement. *BMJ* **350**, (2015).

50. Xiong, J. *et al.* Impact of COVID-19 pandemic on mental health in the general population: A systematic review. *J. Affect. Disord.* **277**, 55–64 (2020).

51. Tromans, S. *et al.* Patterns of use of secondary mental health services before and during COVID-19 lockdown: observational study. *BJPsych Open* **6**, (2020).

52. Hutchings, R. The impact of Covid-19 on the use of digital technology in the NHS. 23.

For peer review only

For peer review only - http://bmjopen.bmj.com/site/about/guidelines.xhtml

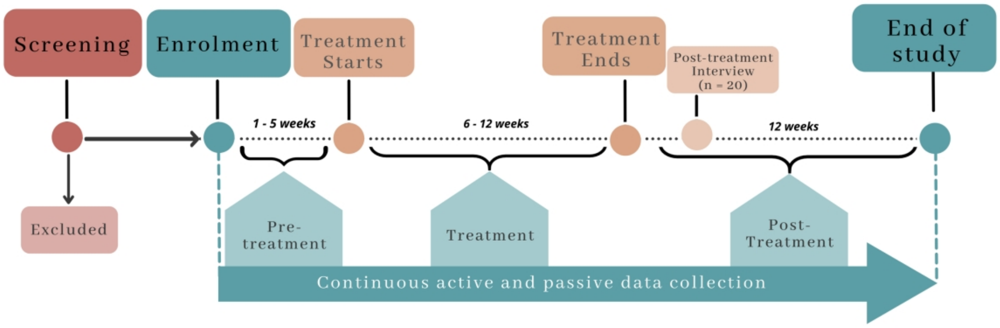

Figure 1. Study timeline for participants.

228x228mm (300 x 300 DPI)

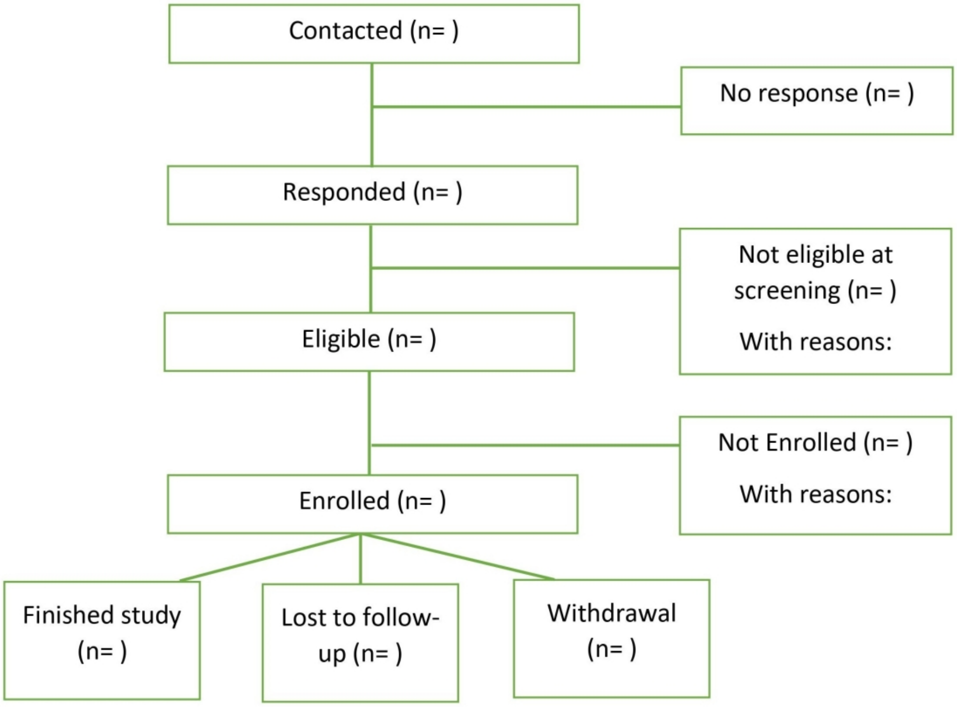

Figure 2. Participant flow in the RAPID study.

181x138mm (300 x 300 DPI)

SUPPLEMENTARY INFORMATION FOR

Using Digital Health Tools for the Remote Assessment of Treatment Prognosis In Depression (RAPID): A Study Protocol for a feasibility study

*Valeria de Angel** [1,2], *Serena Lewis* [1,3], *Sara Munir* [4], *Faith Matcham* [1], *Richard Dobson* [2,5], *Matthew Hotopf* [1,2]

1. Institute of Psychiatry, Psychology and Neuroscience, King's College London, London, UK
2. NIHR Maudsley Biomedical Research Centre, South London and Maudsley NHS Foundation Trust, London, UK
3. Department of Psychology, University of Bath, Bath, UK
4. South London and Maudsley NHS Foundation Trust, London, UK
5. Department of Biostatistics and Health Informatics, Institute of Psychiatry, Psychology and Neuroscience (IoPPN), King's College London, 16 De Crespigny Park, London, SE5 8AF, UK

* Corresponding Author contact details:
E3.08, 3rd floor East Wing, Institute of Psychiatry, Psychology and Neuroscience
16 De Crespigny Park, London, SE5 8AF
E: valeria.de_angel@kcl.ac.uk

For peer review only

Supplementary Note 1 –

Post-Treatment Interview

The primary purpose of this interview is to gain insight into the acceptability of remote assessment and to provide contextual information about participation experiences which may be used to improve future studies using digital sensing technologies.

[TURN ON recording]

Interviewer: "Thank you for agreeing to do the interview. I'd like to ask some questions about your experiences of participating in the study during treatment, specifically, how you found using the technology so the researchers can understand how to deliver a better service in the future. When I talk about participating in the study I mean using the Fitbit, answering Questionnaires on your phone and via email, having the passive app and completing the THINC it app games (the ones about memory and attention). I am keen to understand what worked for you and what **didn't,** so please be completely honest.

This interview is being recorded and should last around 30 minutes, but you can let me know if you would like the recorder to be turned off at any point. All information you provide is anonymous. Information from all the interviews we carry out will be analysed but no identifiable information about you will be included. Also, we might use some quotes from you for academic posters, research papers and a doctoral thesis, but again these will be totally anonymous and not connected to you in any way. Do you have any questions?

This is a semi-structured interview, which means that I have a list of relatively open-ended questions that I will ask you. This means that questions might come up over the course of the interview that you may have already touched on in a previous answer, so If some of the questions seem repetitive or related to your previous answers, please bear with us. We just want to make sure we understand your experience the best we can - thank you!"

A) Participation experience: Identify opportunities for improvement in the study experience (3 mins)

1. How are you finding the experience of participating?
2. Are you finding any aspect of participating in the study particularly burdensome? Can you give me some examples of how/when/why? Anything else that you dislike?

Prompts: [Remind them that this isn't just about the Fitbit] Do you ever feel you needed to spend more time or effort than you would like to? Does taking part cause any disruption to your life?

3. Do you see any personal benefits of participating in the study? Can you give me a few examples of what they are? Anything else that you particularly like.

Prompts: remind them that this isn't just about the Fitbit.

4. Did the study change the way you monitored your health in any way?

B) Working the Study into Daily Life: Identify pain points of fitting the study into daily life (3 mins)

5. Has taking part in this study fit into your usual routine?

Prompts: Did you have to change any aspect of your routine in order to be able to take part? What didn't fit?

For peer review only - http://bmjopen.bmj.com/site/about/guidelines.xhtml

6. Are there any aspects of the study that make it difficult for you to fully participate?

Prompt: Did anything stop you from completing some of the tasks/questionnaires or wear the Fitbit?

7. What could have made it easier for you to participate in this study?
8. Did tracking your behaviour change your behavior?

Prompt: Would you have behaved differently if you had not been wearing a Fitbit, or if you knew your data/responses wouldn't eventually be analysed by a research team.

C) Participation during Psychological Treatment (12 mins)

9. If you have experienced changes in your mood or anxiety levels in the past few months, has it affected the way you have participated in this study? In what way?

Prompts: Has an improvement in mood made you more likely to engage with some of the tasks/questionnaires? Was it easier to remember or motivate yourself to continue?

10. Do you feel like being in this study has had an impact on the psychological therapy you have received? In what way?

Prompt: Has it made you more/less likely to attend sessions, more/less likely to engage with homework. Did tracking your behaviour, sleep, etc impact how you experienced therapy sessions?

11. Did you talk to your therapist about the study? In what way? What about it?

12. Was participating in this study during treatment a positive, negative. neutral experience for you? In what way?

13. If so, what sorts of things do you think could be done to increase any positive impact it has on your treatment.

Prompt: Is there any way in which the wearables or questionnaires could be used in a way which complements therapy within IAPT?

14. What could be done to decrease any negative impact on your treatment?
15. You've been in the study before, during, and after treatment. Was it easier/harder to participate in the study during treatment than outside of it?

16. Do you feel like tracking your behaviour, mood, sleep has had an impact on your mental health?

17. How did you feel about receiving treatment remotely (via telephone or video call)?

Prompt: was it easy/difficult to communicate? Were you comfortable with the technology? Was it convenient?

D) Experience with Apps and wearables: Assess familiarity, learning curve, pain points (4 mins)

18. Had you ever used health tracking apps before coming into the study? What for?

Prompts: by health apps I mean apps that you use to measure or help with physical health/activity or mental health, e.g., Strava, headspace other anxiety apps, food intake, dairies, etc.

19. Were you familiar with wearable devices (activity tracker, health tracker, etc.) prior to this study?

Prompts: What type of device was it? How long did you use it? If you stopped using it, why did you stop?

For peer review only - http://bmjopen.bmj.com/site/about/guidelines.xhtml

20. Was there anything else you would have liked to keep track of during treatment?
Prompts: anything to do with physical health, or emotional/mental health, diaries, etc.

21. Do you ever have any difficulties using the device? Can you give me some examples?
Prompts: At night, for exercise, During charging? During syncing? During setup? Were you able to wear the device for long periods of time or whilst you were sleeping without it causing any annoyance or discomfort?

22. Have you ever chosen not to wear the device? Why was that, and why/when did you start wearing it again?
Prompts: Did level of comfort impact on your choice to wear the device?

23. Are you ever concerned about what people thought of you wearing the device? Can you give me an example?
Prompts: Did it make it easier or harder for you to take part in this study? Did it make you more or less concerned about what others would think about you using the smartphone or wearable device?

24. Because of the pandemic and the social distancing measures taken by the government, do you feel differently about using smartphones and wearable devices? Can you tell me why?
Prompt: do you feel more, or less, inclined to use and engage with your smartphone or wearable devices?

E) Data Collection, Privacy and Sharing (4 mins)

25. How did you feel about the data being collected passively/automatically, without you needing to interact with the device?
Prompt: Did you like this more or less than when you had to interact with the device, e.g. when you had to complete a survey?

26. How would you feel about the information collected during the study going to your therapist or healthcare provider?
Prompts: What are your thoughts about the data being automatically available to your GP or healthcare provider? Would you like to be able to choose what information they receive?

27. Would it have been useful for you to receive information about the data collected throughout the study? Why or why not?
Prompts: Would it make you more or less likely to participate in a future study? If you would look at feedback, how would you like to receive this? Should it be optional? Provided in the app? Should it be visual, with graphs, or verbal feedback? What kinds of graphs are useful? How often should it be provided?

28. Did you 'feel monitored'?
Have the impression of being watched or that your movements were being tracked, monitored.

F) Closing & Improvements needed in Study Design (1 mins)

29. Do you think there is value in this approach to gathering data about your health? How would you describe that value?

30. Is there anything else you would like to say?

For peer review only
